# Identification of ALKBH6 as a nucleotide demethylase with a distinct substrate preference

**DOI:** 10.1016/j.jbc.2025.110638

**Published:** 2025-08-28

**Authors:** Susmita Das, Sourbh Rankawat, Unnikrishnan P. Shaji, Nikhil Tuti, Nafeesa Shahnaz, Sandipan Ray, Roy Anindya

**Affiliations:** Department of Biotechnology, Indian Institute of Technology Hyderabad (IITH), Sanga Reddy, Telangana, India

**Keywords:** methylating agents, direct repair, *AlkB*, DNA, nucleotide, demethylation, ALKBH6

## Abstract

AlkB homolog (ALKBH) family enzymes remove the methyl groups from a variety of substrates including ssDNA and dsDNA, RNA, and proteins. There are eight different ALKBH genes (ALKBH1–8) encoded in the human genome. However, the identity of the substrate of ALKBH6 has remained elusive. In this study, through biochemical experiments and mass spectrometry analysis, we demonstrate that ALKBH6 effectively demethylates *N*-7-methyl-GMP, and *N*-1-methyl-adenosine monophosphate. We observed that the presence of a phosphate group in the substrate molecule is essential for ALKBH6 activity as it showed no activity toward methylated bases or the methylated nucleosides. Enzyme kinetic analysis led to the identification of the catalytic and substrate binding residues of ALKBH6 and revealed that succinate and oncometabolite 2-hydroxyglutarate can act as inhibitors of this enzyme. By using mass spectrometry analysis, immunofluorescence microscopy, and ELISA, we confirmed that *N*-7-methyl-GMP and *N*-1-methyl-AMP are also the endogenous substrates of ALKBH6. This functional characterization of ALKBH6 broadens the substrate range of ALKBH family proteins and positions ALKBH6 as one of the key players involved in sanitizing the *N*-methylated nucleotides.

Endogenous and exogenous methylating agents induce a variety of DNA adducts, including N-methylated DNA bases (1, 2). Methylation of guanine N-7 is the most abundant methylated site in DNA when treated with common methylating agents, such as methyl methanesulfonate (MMS). Some of the other N-methylated adducts include *N-*1-methyladenine (1me-A) and *N-*3-methylcytosine (3me-C). These adducts are considered cytotoxic as they disrupt hydrogen bonding between base pairs and hinder DNA replication (3). N-methylated bases can occur in RNA as well, either as reversible posttranscriptional modification or following exposure to methylating agents, such as MMS. Guanosine N7 methylation is present in the 5′-caps and also occurs internally in mRNAs and miRNAs (4, 5), tRNAs and 18S rRNAs (6). 3me-C is present in tRNA (7, 8, 9), and 1me-A is also predominantly present in tRNA and limited amounts in rRNA and mRNA of eukaryotic cells (10).

*Escherichia coli* AlkB and certain human AlkB homolog (ALKBH) proteins primarily repair 1me-A and 3me-C in DNA (11, 12, 13, 14, 15). It was reported that ALKBH2 and ALKBH3 preferentially repair dsDNA and ssDNA, respectively (15, 16, 17). Deoxy-7me-G is unstable and spontaneously removed from DNA in a neutral aqueous solution (18). Alternatively, it is removed by methylpurine DNA glycosylase which catalyzes the excision of base substrates, followed by multistep base excision repair (19). The stability of 7meG adducts in RNA is comparatively high and unlike deoxy-7me-G and is not spontaneously removed (20, 21). AlkB family members are not known to repair 7me-G from DNA or RNA.

One of the ALKBHs, whose substrate is not known yet, is ALKBH6. This protein shares structural similarities to ALKBHs, including the double-stranded β-helix fold at the catalytic core and nucleotide recognition lid (22). Three conserved amino acid residues, namely, H114, D116, and H182 comprise the catalytic center and stably coordinate the Fe(II) ion and 2-oxoglutarate (2OG) (cosubstrate). Heterologous expression of ALKBH6 in Alkb-deficient *E. coli* strain was reported to suppress MMS-induced cytotoxicity and silencing of ALKBH6 in pancreatic cancer cell lines was shown to promote cell survival following exposure to alkylating agents (23). However, recombinant ALKBH6 did not exhibit any activity when methylated DNA or RNA was used as a substrate (24). Analysis of the structure of the ALKBH6 catalytic pocket revealed a narrow positively charged groove on the surface in contrast to ALKBH3 and ALKBH5 (22), indicating that ALKBH6 may not directly repair DNA or RNA (22). Based on this structural insight, we hypothesized that ALKBH6 might possess the capability to demethylate nucleotides *via* oxidative demethylation. We demonstrate here that ALKBH6 can demethylate various N-methylated nucleotides, including 7me-GMP/7me-dGMP and 1me-AMP/1me-dAMP, but not the corresponding methylated nucleoside or methylated bases. Enzyme kinetic analysis revealed that catalytic residues H114, D116, and H182 are essential for the activity and binding analysis revealed that the Y120 residue is vital for interaction with the substrate. By knocking down ALKBH6 expression, we confirm that 7me-GMP and 1me-AMP are also the endogenous substrates of ALKBH6.

## Results

### ALKBH6 is a functionally active Fe(II)/2OG-dependent dioxygenase

**Figure 1:**
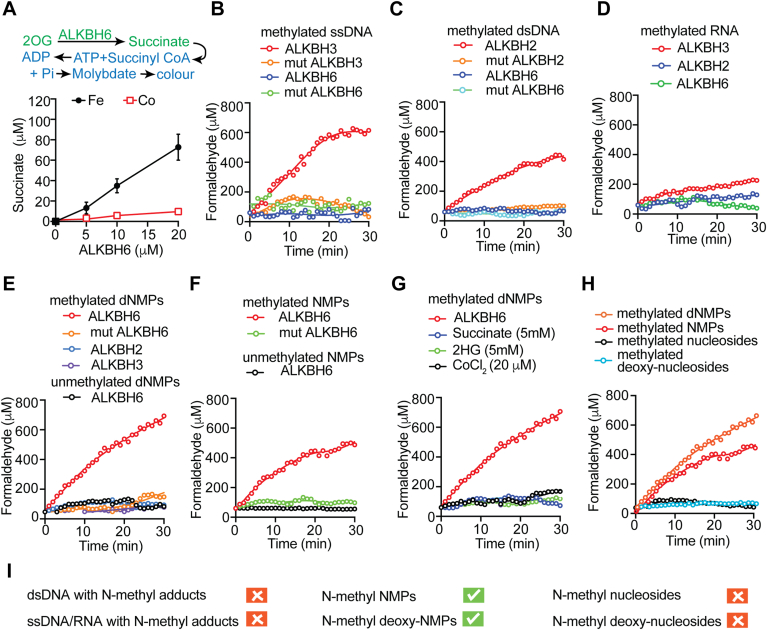
**Identification of substrate of ALKBH6.***A*, uncoupled turnover of 2OG catalyed by increasing concentration of ALKBH6. Error bar represent mean SE (*n* = 4). *B*, ALKBH6 could not demethylate oligonucleotides (ssDNA) substrate. ALKBH3 was used as positive control. Catalytically dead mutant ALKBH6 (mut ALKBH6, H114A, D116A, H182A) and ALKBH3 (mut ALKBH3 H191A, D193A, H257A), were used as negative control. *C*, ALKBH6 could not demethylate duplex oligonucleotides (dsDNA) substrate. ALKBH2 was used as positive control. Catalytically dead mutant ALKBH6 (mut ALKBH6) and ALKBH2 (mut ALKBH2 H171A, D173A) were used as negative control. *D*, ALKBH6 could not demethylate ssRNA as substrate. ALKBH2 and ALKBH3 were used as control. *E*, ALKBH6 could demethylate deoxy-NMPs as substrate but ALKBH2 and ALKBH3 could not. As negative control, mutant ALKBH6 and unmethylated deoxy-NMPs were used. *F*, ALKBH6 could demethylate NMPs as substrate. As negative control mut ALKBH6 and unmethylated deoxy-NMPs were used. *G*, ALKBH6-mediated demethylation of NMPs was inhibited by 2OG mimetic compounds (succinate and 2-hydroxyglutarate) or Co(II) that competes with Fe(II) for binding to the catalytic centre. *H*, ALKBH6 could not demethylate ribonucleosides or deoxy-ribonucleosides as substrate. Methylated NMP and deoxy-NMPs were used as positive control. All data represent mean (*n* = 5). *I*, schematic summary of the result. Here, ‘√’ represents substrates demethylated by ALKBH6 and “X” indicates nonsubstrate. Additional results are in Fig. S1. 2OG, 2-oxoglutarate; ALKBH, AlkB homolog; deoxy-NMP, deoxy-nucleotide monophosphate.

All active Fe(II)/2OG-dependent dioxygenases display uncoupled decarboxylation of 2OG to succinate. The rate of the uncoupled reaction was reported to be slower than the substrate-based reaction, but it provided proof of catalytic activity, nonetheless (25). This uncoupled reaction was observed in all the catalytically active human ALKBH proteins, including ALKBH2 (26), ALKBH3 (26), ALKBH5 (27), and ALKBH8 (28). To investigate the catalytic activity of ALKBH6, we first studied its uncoupled 2OG turnover. We employed a one-step colorimetric assay using succinyl-CoA synthetase (*E coli* SucCD) enzyme (29). The conversion of succinate to succinyl-CoA is accompanied by the hydrolysis of ATP to form ADP and orthophosphate. The latter was quantified colorimetrically using a molybdenum reagent (Fig. 1*A*). When increasing concentrations of WT ALKBH6 were incubated with the cosubstrate 2OG without any substrate, succinate was produced in a dose-dependent manner (Fig. 1*A*). When a divalent catanionic inhibitor, such as cobalt chloride, which prevents Fe(II) binding was added to the reaction mixture, uncoupled succinate production was inhibited (Fig. 1*A*). Overall, this uncoupled succinate production by ALKBH6 indicated that ALKBH6 is a catalytically active enzyme.

### N-methylated dNMPs and NMPs are the primary substrates of ALKBH6

Uncoupled succinate production by ALKBH6 prompted us to test the demethylation activity of ALKBH6. When Fe(II)/2OG-dependent dioxygenases oxidise N-methyl-adducts to the hydroxymethyl group, an unstable intermediate is formed, which is later nonenzymatically released as formaldehyde (30). We first tested methylated oligonucleotides (ssDNA) as a substrate for ALKBH6, using ALKBH3 as a positive control and monitored the reaction by formaldehyde production formaldehyde dehydrogenase (FDH) (17, 31, 32, 33). As shown in Figure 1*B*, no significant demethylation was detected with ALKBH6. Next, we examined whether ALKBH6 can remove the methyl group from the methylated duplex oligonucleotides (dsDNA). Catalytically dead mutants of ALKBH6 (H114A, D116A, H182A) and ALKBH2 (H171A, D173A) were used as the negative controls and WT ALKBH2 was used as the positive control. Like methylated ssDNA, ALKBH6 could not repair methylated dsDNA (Fig. 1*C*). As expected, ALKBH3 and ALKBH2 activity resulted in the release of formaldehyde, suggesting successful DNA repair (Fig. 1, *B* and *C*). We also examined if ALKBH6, ALKBH2, and ALKBH3 could repair methylated RNA (Fig. 1*D*); however, we observed only trace amounts of formaldehyde release in the presence of these enzymes. Next, we wanted to know whether ALKBH6 could demethylate deoxy-nucleotide monophosphates (dNMPs). To examine this, methylated dNMPs (me-dNMPs) were used as the substrate for ALKBH6. A significant formaldehyde release was observed, suggesting ALKBH6-catalyzed demethylation of me-dNMPs (Fig. 1*E*). Incubation of me-dNMPs with catalytically dead mutant ALKBH6 did not result in any formaldehyde production, confirming the specificity of ALKBH6-catalyzed formaldehyde release (Fig. 1*E*). Similarly, no formaldehyde production was detected in the presence of unmodified dNMPs (Fig. 1*E*). Incubation with me-dNMPs with ALKBH3 and ALKBH2 did not produce any formaldehyde, suggesting the specificity of the ALKBH6-catalyzed reaction (Fig. 1*E*). The unexpected demethylation of dNMP by ALKBH6 made us hypothesize that ALKBH6 might also act on methylated nucleotide monophosphates (me-NMPs). To test this hypothesis, me-NMPs were used as the substrate for ALKBH6. Again we observed a significant formaldehyde release, suggesting that ALKBH6 can also catalyze the demethylation of me-NMPs (Fig. 1*F*). Unmodified NMPs or the use of catalytically inactive mutant ALKBH6 did not result in any formaldehyde release, suggesting that ALKBH6-mediated formaldehyde release was specific (Fig. 1*F*). We did not detect any formaldehyde production by ALKBH2 or ALKBH3 when methylated NMP was used as substrate (Fig. S1). Since all members of AlkB family of oxidative demethylases are known to be inhibited by high succinate concentration, we investigated whether ALKBH6 activity is also inhibited by succinate. As shown in Figure 1*G*, increasing the concentration of succinate indeed inhibited ALKBH6 activity. Notably, to inhibit the *in vitro* reaction, a millimolar concentration of succinate was required. It is not known whether the intracellular concentration of succinate (1–5 μM) (34) would be inhibitory for the activity of this enzyme. Replacing the active site Fe(II) ion at the catalytic center with other transition metal ions inhibits several Fe(II)/2OG family enzymes such as JMJD2A, JMJD2E, PHD2, ALKBH2, and ALKBH3 (35, 36). Indeed, when Co(II) was added to the reaction, ALKBH6-mediated demethylation of me-dNMPs was completely inhibited (Fig. 1*G*). Accumulation of high concentration of 2-hydroxyglutarate (2HG) was reported to inhibit 2OG-dependent dioxygenases because 2HG is a competitive inhibitor of enzymes requiring 2OG as a cofactor, including TET DNA demethylases (TET1/2/3) (37, 38), histone lysine demethylase JmjC (39), and AlkB family of dioxygenases (40). Since ALKBH6 activity is also inhibited by the 2HG, we incubated ALKBH6 with me-dNMPs in the presence of 2HG. As shown in Figure 1*G*, 2HG inhibited the demethylation activity of ALKBH6. We also examined if ALKBH6 can remove methyl groups from the methylated nucleosides. When 5′-phosphate was removed from me-NMPs and me-dNMPs using *E coli* UshA 5′-nucleotidase, ALKBH6 could not demethylate and formaldehyde release was not observed (Fig. 1*H*). These findings collectively indicate that methylated nucleosides are also not the substrate for ALKBH6. Only me-NMPs and me-dNMPs with the 5′-phosphate group serve as the substrate for ALKBH6 (Fig. 1*I*).

### ALKBH6 effectively demethylates 7meGMP, 3meCMP, and 1meAMP

**Figure 2:**
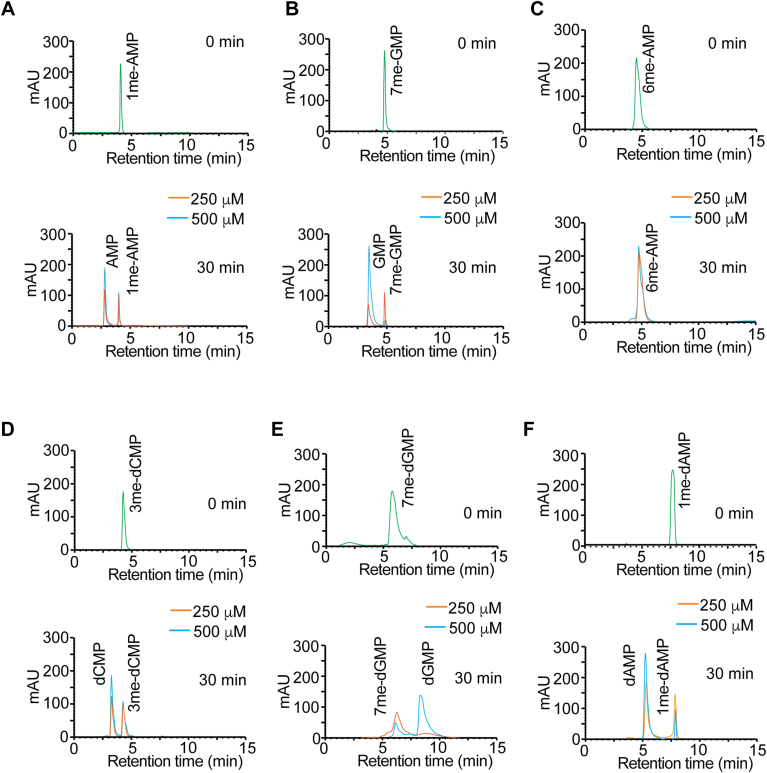
**Demethylation activity of ALKBH6.** HPLC analysis was carried out before and after ALKBH6-mediated demethylation. HPLC chromatogram of (*A*) 1me-AMP, (*B*) 7me-GMP, (*C*) 6me-AMP, (*D*) 3me-dCMP, (*E*) 7me-dGMP, and (*F*) 1me-dAMP. Results with two different substrate concentration (250 and 500 μM) were analyzed. Notably, the higher substrate concentration yielded more products because the product formation is incomplete within the 30-min time frame and continued at a rate proportional to the substrate concentration. For additional substrate concentration of reaction depicted in *A* and *B*, please see Fig. S8, *A* and *B*. ALKBH, AlkB homolog.

**Figure 3:**
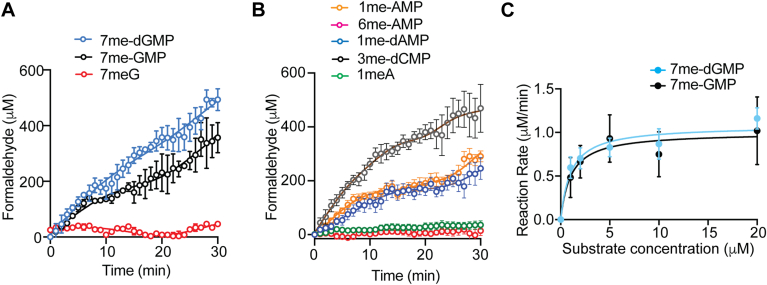
**Enzyme kinetics of nucleotide demethylation by ALKBH6.** Time courses of averaged product (formaldehyde) formation in the presence of (*A*) 7me-GMP and 7me-dGMP, (*B*) 1me-AMP, 6me-AMP, 1me-dAMP, and 3me-dCMP. 7me-Guanosine and 1me-Adenosine were used as negative controls. *C*, Michaelis–Menten plots of ALKBH6. Data in Figures (*A*), (*B*), and (*C*) represent mean ± SE (error bars) from seven biologically independent experiments (n = 7), each with 3 replicates. ALKBH, AlkB homolog.

To get a better understanding of the substrate specificity of ALKBH6, we next evaluated whether ALKBH6 directly removed the methyl group from the specific nucleotides by resolving the nucleotides using reversed-phase HPLC. We analyzed methylated NMPs, including 1me-AMP, 6me-AMP, and 7me-GMP and deoxy-NMPs, including, 1me-dAMP, 3me-dCMP, and 7me-dGMP. The HPLC was calibrated with standard GMP/dGMP, AMP/dAMP, and dCMP to allow identification of the elution position of each nucleotide (Fig. S7). When incubated with purified recombinant ALKBH6, 1me-AMP, and 7me-GMP were converted to AMP and GMP, respectively (Fig. 2, *A* and *B* and S8). Notably, the higher substrate concentration produced more products indicating that the reaction was in the initial phase of an enzyme-catalyzed reaction within the time frame (30 min). We did not observe any demethylation of 6me-AMP suggesting that 6me-AMP may not be a substrate of ALKBH6 (Fig. 2*C*). ALKBH6 could efficiently demethylate deoxy-nucleotides, including 3me-dCMP (Fig. 2*D*), 7me-dGMP (Fig. 2*E*), and 1me-dAMP (Fig. 2*F*). We wanted to further confirm whether ALKBH6-mediated demethylation of these me-NMPs/me-dNMPs resulted in the release of formaldehyde. For this, FDH-coupled indirect enzyme assay was used which allowed steady-state formaldehyde measurement. Formaldehyde release was observed for 7me-GMP and 7me-dGMP (Fig. 3*A*) as well as 3me-dCMP and 1me-dAMP (Fig. 3*B*). When 7me-GTP and 1me-ATP were used as substrates, the rate of formaldehyde production was comparatively less (Fig. S3, *A*–*D*). This result suggests that among different nucleotides, monophosphates could be preferentially demethylated by ALKBH6. Interestingly, we did not observe any demethylation when 6me-AMP was used as substrate (Fig. 3*B*). As expected, ALKBH6 could not demethylate nucleosides 7me-guanosine or 1me-adenosine (Fig. 3, *A* and *B*).

To confirm that demethylation of 7me-GMP and 1me-AMP was specifically due to the catalytic activity of ALKBH6, we used a triple catalytic mutant of ALKBH6 (H114A, D116A, H182A) as a negative control. This mutant showed complete loss of activity (Fig. S3, *E* and *F*). To exclude the possibility that the triple mutant might affect protein folding, we generated a single catalytic mutant targeting one of the catalytic residues (H182A) (Fig. S2). The purified ALKBH6-H182A protein also exhibited loss of demethylase activity, comparable to the triple mutant (Fig. S3, *E* and *F*), confirming that the loss of activity is due to catalytic site disruption.

As methylated guanine nucleotides showed the maximum formaldehyde production, we determined the kinetic parameters of ALKBH6 using 7me-GMP and 7me-dGMP as the substrates and *K*_*M*_ and *k*_cat_ values were calculated from Michaelis–Menten plots of the data (Fig. 3*C*). ALKBH6 exhibited similar K_M_ for 7me-GMP and 7me-dGMP (1.03 μM) and *k*_cat_ (10.8 min^−1^) with 7me-dGMP and 7me-GMP (10 min^−1^). K_M_ and k_cat_ of ALKBH6 can be compared to typical nudix sanitizing enzymes (41). Interestingly, the K_M_ of ALKBH6 was found to be less compared to the reported K_M_ for O6-methyl-dGTP of MTH1 (13.2 μM) (42). The k_cat_ of ALKBH6 was also lower than that of several Nudix hydrolase sanitizing enzymes (41), including MTH1, which has been reported to exhibit k_cat_ values of 8.3 s^−1^ for O^6^-methyl-dGTP and 0.3 s^−1^ for O^6^-methyl-GTP (42). Demethylation activity against 1me-AMP/1me-dAMP was also identified in the *E coli* AlkB enzyme earlier (43). AlkB displayed *K*_M_ of 2 μM and *k*_cat_ of 4.2 min^−1^ with 1me-dAMP (41), whereas K_M_ of ALKBH6 for 1me-AMP was 0.96 μM and K_cat_ 10.8 min^−1^. The k_cat_ of ALKBH6 was comparatively higher than the other ALKBH enzymes involved in DNA repair (40). For example, the k_cat_ of ALKBH2 for 1-meA has been reported to be in the range of 1.1 to 2.5 min^−1^, whereas ALKBH3 demonstrated a k_cat_ of approximately 1.7 min^−1^ for 3meC (40). These results suggest that ALKBH6 is efficient in removing methyl groups from various methylated nucleotides and the kinetic parameters are comparable to similar human enzymes identified before.

### Confirmation of demethylated products by LC/MS analysis

**Figure 4:**
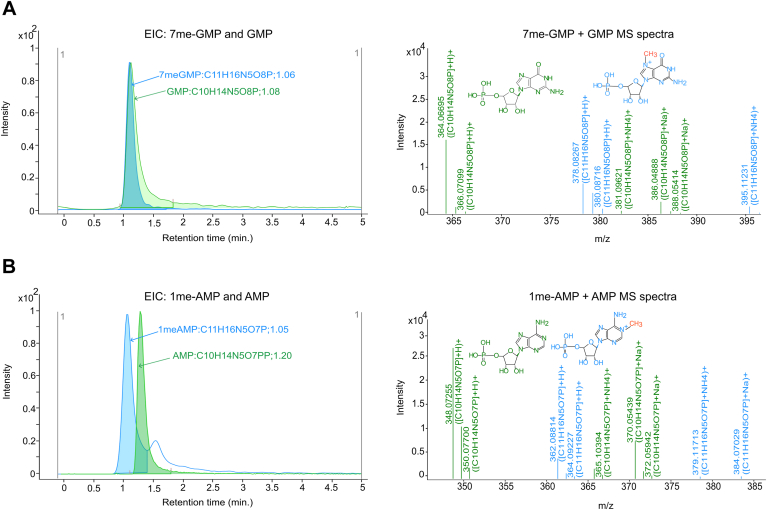
**Mass spectrometric analysis of ALKBH6-mediated demethylation of nucleotides.** Overlaid extracted ion chromatograms (EICs, *top*) and corresponding mass spectrometry (MS) spectra (*bottom*) illustrate coelution profiles over time (*x*-axis) and intensity (*y*-axis). *A*, GMP (*green*) and 7me-GMP (*blue*) in the reaction mixture (7me-GMP + GMP). The reference MS spectra indicate the *m/z* values for 7me-GMP (378.08) and GMP (364.06) in reaction 1. *B*, 1me-AMP (*blue*) and AMP (*green*) in the reaction mixture (1meA + AMP). The reference MS spectra indicate the *m/z* values for 1me-AMP (362.08) and AMP (348.07) in reaction 2. ALKBH, AlkB homolog.

Samples were separated by ultra-high performance liquid chromatography and analyzed using LC/MS to confirm the demethylation of nucleotides following ALKBH6 treatment. Extracted wavelength chromatograms at 260 nm revealed coelution of 7me-GMP and GMP in the first reaction mixture at a retention time (RT) of 1.06 min (Fig. S9). A similar coelution pattern was observed for 1meAMP and AMP in the second reaction at RT 1.04 min (Fig. S9). LC/MS analyses provided distinct RTs and mass confirmations for the demethylated products with identification scores >95 and were verified with the reference standards (Fig. S10). In the first reaction mixture, 7me-GMP was detected at 378.08 *m/z*, 1.06 min RT and GMP was confirmed at 364.06 *m/z*, 1.08 min RT (Fig. 4*A*). Consistent with demethylation, the mass spectrum of the first reaction showed a higher intensity peak for GMP (364.06 *m/z*, 1.08 min RT) than 7me-GMP (378.08 *m/z*, 1.06 min RT) (Fig. 4*A*). Similarly, the second reaction displayed a higher intensity peak for AMP (348.07 *m/z*, 1.20 min RT) than 1meAMP (362.08 *m/z*, 1.05 min RT) (Fig. 4*B*). We performed a series of additional mass spectrometric experiments for multiple controls, including reactions with mutant ALKBH6 and reactions with WT ALKBH6 in the absence of the cofactor Fe^2+^ and substrate 2OG. Notably, no demethylated products (GMP or AMP) were detected in these control experiments (Fig. S11). We compared the identified reaction substrates and products with the respective standards using their *m/z* values (mass tolerance ±5 ppm) for robust confirmation of substrate and product identities (Fig. S10). We also evaluated the demethylation of deoxyribonucleotides (7me-dGMP and 1me-dAMP) by ALKBH6. LC/MS analysis confirmed the presence of the demethylated products, dGMP with a mass of 348.078 *m/z* and an identification score of 99.8, and dAMP with a mass of 332.07 *m/z* and an identification score of 99.62, respectively. However, the substrates (7me-dGMP and 1me-dAMP) were not detected in the mass spectra, likely due to their rapid conversion to dGMP and dAMP, as suggested by the coelution observed in the diode array detector (DAD) chromatogram (Fig. S12).

### Identification of the key amino acid residues for ALKBH6-catalyzed demethylation

**Figure 5:**
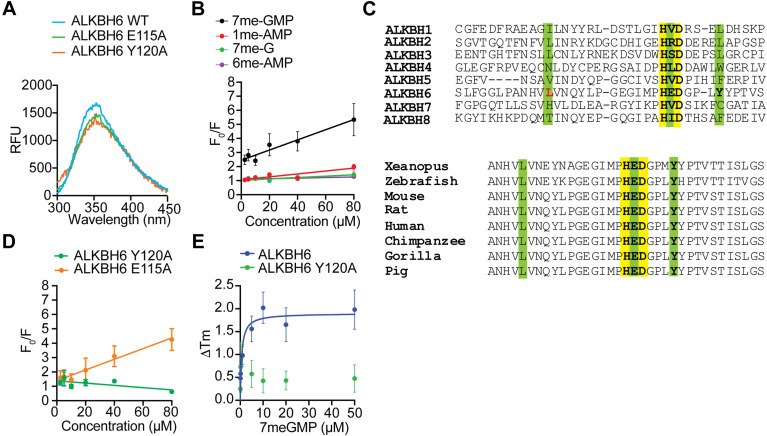
**Identification of the ALKBH6 amino acid residues involved in the interaction with the methylated nucleotide substrate.***A*, the graphs show averaged fluorescence emission spectra of recombinant WT ALKBH6 and the two mutants (Y120A and E115A). *B*, plot of Trp fluorescence titrations performed with 7me-GMP, 1me-AMP, 6me-AMP, and 7me-guanosine. Titration data were represented by dividing the measured fluorescence in the presence of inhibitors (F) by the fluorescence measured in the absence (F_0_). *C*, sequence alignment of human ALKBH proteins (*top*) and ALKBH6 proteins from different organisms. The conserved catalytic site residues (*yellow*) and modified nucleotide interacting residues (*green*) as predicted by molecular docking (supplementary information) are also shown. *D*, plot of Trp fluorescence titrations using increasing concentration of 7me-GMP and ALKBH6-E115A and ALKBH6-Y120A. All the data were calculated with best-fit parameter values and 95% confidence intervals (CIs) in GraphPad-Prism software. *E*, the thermal melting profile of ALKBH6 and ALKBH6-Y120A was monitored using SYPRO Orange fluorescent dye in the presence of 7me-GMP and the plot of the transition melting temperature shift (ΔT_m_) was assessed with increasing 7me-GMP concentrations. Data in Figures (*D*) and (*E*) represent mean ± SE (error bars) from five biologically independent experiments (n = 5), each having 3 replicates. ALKBH, AlkB homolog.

The sensitivity of the fluorescence emission of the tryptophan residue on its environment allowed us to examine the binding of methylated nucleotides to ALKBH6. 2OG was omitted in the binding reaction to avoid ALKBH6-mediated demethylation. ALKBH6 contains three Trp residues (W50, W61, and W78) and recombinant ALKBH6 exhibited a tryptophan emission maximum at ∼356 nm and showed quenching in the presence of 7me-GMP (Fig. 5*A*). A similar result was observed in the presence of 1me-AMP; however, the amount of quenching was less. These results indicate that the presence of 7me-GMP reduced solvent accessibility to ALKBH6. Stern–Volmer constants (*K*_*sv*_) for the 7me-GMP (0.036 ± 0.009 μM^−1^) match with the relative quenching of their tryptophan fluorescence (Fig. 5*B*). As expected, the *K*_*sv*_ value of 1me-AMP (0.011 ± 0.002 μM^−1^) was found to be less suggesting the lower quenching of their tryptophan fluorescence (Fig. 5*B*). The ALKBH6 structure revealed that the catalytic center of ALKBH6 contains a Fe(II) metal coordinated to the H114, D116, and H182 (22). Interestingly, the glutamate residue (E115) is located between the Fe(II)-interacting residues H114A and D116A. Apart from these residues, a few other residues of the active site of ALKBH6 are in the equivalent position of ALKBH2, *viz.*, L58, N60, L101, and Y120 of ALKBH6 and Q112, T114, L157, L178 of ALKBH2, respectively (44). From the sequence comparison, it is clear that Glu 115 and Tyr 120 of ALKBH6 are conserved across vertebrate species (Fig. 5*C*) (22). We also carried out molecular docking and verified that the substrate 7me-GMP and 1me-AMP could be positioned within the catalytic site without any steric clash (Fig. S5). Model also showed distinct amino acid residues interacting with both the methylated nucleotides, 7me-GMP and 1me-AMP (Table S3). Structural studies indicate that for efficient demethylation by AlkB family proteins, the methyl group of the substrate must be positioned within approximately 3.5 to 4 Å of the Fe(II) atom in the catalytic site. This spatial arrangement is essential to facilitate hydroxylation of the methyl group, enabling its removal as formaldehyde. As shown in Fig. S5, the distance between the metal ion of the ALKBH6 structure and methyl carbon of 7me-GMP and 1me-AMP is less than 4 Å. Interestingly, the model showed that the methyl group of 6me-AMP is positioned approximately 9 Å from the metal center, which likely explains why it is not efficiently demethylated by ALKBH6 *in vitro*.

**Figure 6:**
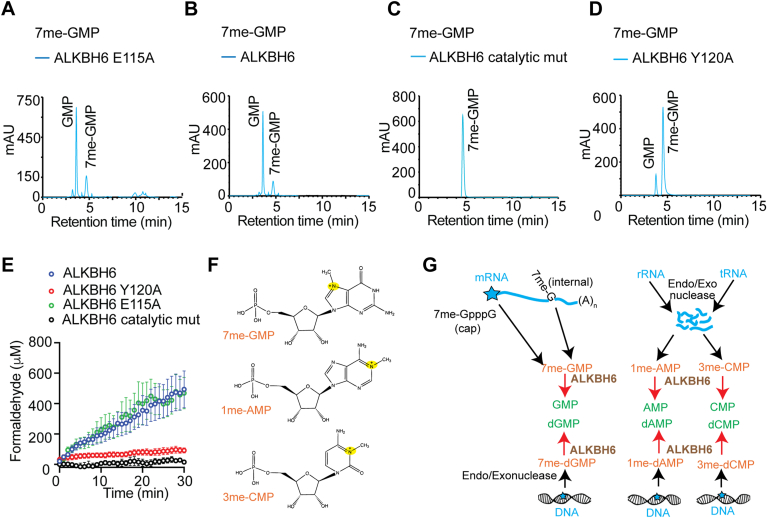
**Mutation affecting substrate binding of ALKBH6 diminishes the demethylation activity.** HPLC analysis was carried out to monitor the demethylation using (*A*) ALKBH6-E115A (*B*) ALKBH6 (*C*) catalytically dead ALKBH6 and (*D*) ALKBH6-Y120A. *E*, time courses of formaldehyde formation in the presence of 7me-GMP by ALKBH6 Y120A, and ALKBH6 E115A. Catalytically dead ALKBH6 was used as a negative control and WT ALKBH6 was used as positive control. All the results are obtained using 7me-GMP (500 μM) as substrate. Data represent mean ± SE (error bars) from ten biologically independent experiments (n = 10), each having 3 replicates. *F*, ALKBH6 substrates identified in this study, including 7me-GMP, 3me-CMP, and 1me-AMP, the leaving methyl-group is attached to a positively charged tetravalent nitrogen. *G*, pathways depicting the enzymes involved in removal of 7me-G from mRNA and 1me-A and 3me-C from tRNA, rRNA, and DNA in human cells. Mechanisms of elimination of 7me-GMP, 1me-AMP, and 3me-CMP by ALKBH6 identified in this study are indicated (in *red arrow*). ALKBH, AlkB homolog.

To know if any of these amino residues are involved in substrate binding, we analyzed the effect of 7me-GMP on the quenching of ALKBH6 E115A and ALKBH6 Y120A mutant. *K*_*sv*_ value for ALKBH6 E115A (0.035 ± 0.008 μM^−1^) was approximately similar to WT ALKBH6 (0.036 ± 0.009 μM^−1^), indicating no change in quenching of their tryptophan fluorescence (Fig. 5*D*). However, the *K*_*sv*_ value for ALKBH6 Y120A was found to be negative, indicating a lack of tryptophan fluorescence quenching (Fig. 5*D*). *K*_*sv*_ values for 7me-GTP (0.007 μM^−1^) and 1me-GTP (0.004 μM^−1^) were also found to be much lower than 7me-GMP (0.036 μM^−1^), suggesting poor binding (Fig. S4). Next, differential scanning fluorimetry (DSF) studies using SYPRO Orange were carried out to determine the binding of AlkBH6 and 7me-GMP (Fig. 5*E*). When 7me-GMP binding to the ALKBH6 was examined by DSF, transition melting temperature (*T*_*m*_) shift (ΔT_m_) was observed suggesting that 7me-GMP could increase the stability of ALKBH6 structure and resist thermal denaturation in a concentration-dependent manner. The ΔT_m_ was plotted with the increasing concentration of 7meGMP (ligand) to obtain an apparent dissociation constant of 0.62 μM. When ALKBH6-Y120A was examined by DSF, no significant ΔT_m_ shift was observed (Fig. 5*E*). This DSF binding data are in agreement with our tryptophan fluorescence experiments and confirms the role of Tyr120 residue in the binding of 7meGMP substrate to ALKBH6. We further analyzed the effect of E115A and Y120A mutation on the activity of ALKBH6 using the two methods described before. Catalytically inactive ALKBH6 was also used as a negative control. When the demethylation of 7meGMP was analyzed over time using an indirect FDH-coupled assay, the activity of the E115 mutant was comparable to WT ALKBH6 (Fig. 6, *A* and *B*). However, HPLC analysis of the end product revealed little demethylation of 7me-GMP and production of GMP by ALKBH6-Y120A and a catalytically dead ALKBH mutant (Fig. 6, *C* and *D*). Altogether, these data corroborate our binding studies (Fig. 5) and confirm that Y120 might play an important role in 7meGMP binding.

### Identification of endogenous ALKBH6 substrate

**Figure 7:**
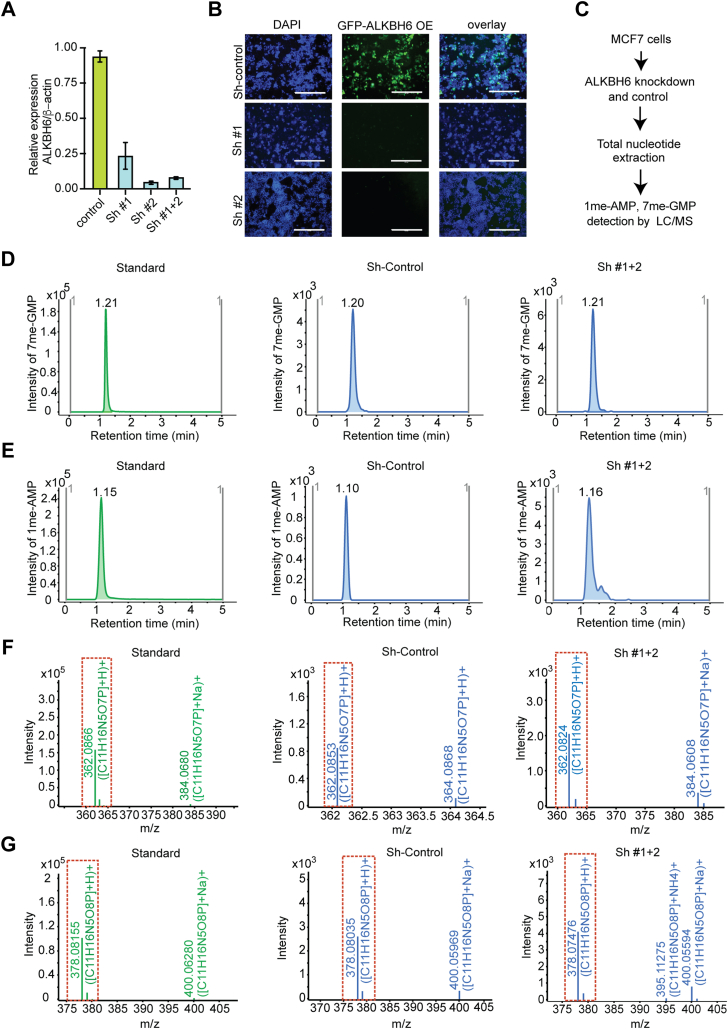
**ALKBH6 knockdown increases endogenous 7me-GMP and 1me-AMP level.***A*, assessment of ALKBH6 gene knockdown. MCF7 cells were transformed with the plasmid vector encoding shRNA to ALKBH6 (Sh #1 and Sh #2), either individually or combined or with just the vector (Sh-Control). Approximately 48 h after transfection, knockdown efficiency was examined by qPCR amplification of ALKBH6. While Sh #1 reduced ALKBH6 expression by 75%, Sh #2 yielded more than 90% knockdown. The expression of ALKBH6 was normalized against that of housekeeping gene β-actin and compared to Sh-control. *B*, MCF7 cells were transfected with plasmid vector encoding Sh #1 and Sh #2, either individually or together (Sh #1 + 2) combined with EGFP-ALKBH6 overexpressing (GFP-ALKBH6 OE) plasmid. ALKBH6 knockdown was assessed by fluorescence microscopy 48 h after transfection. The scale bar represents 400 μm. *C*, schematic presentation of the experiment described in *panel D-G*. *D* and *E*, LC/MS analysis of ALKBH6-mediated demethylation of 7me-GMP and 1me-AMP in MCF7 cell lines. Extracted ion chromatogram (EIC) elution profiles of 7me-GMP and 1me-AMP standards, along with their endogenous levels in control and knockdown (Sh #1 + 2) MCF7 cells, are shown. The *x*-axis denotes retention time (minutes), and the *y*-axis represents the signal intensity for 7me-GMP or 1me-AMP. *F* and *G*, MS spectra of 7me-GMP and 1me-AMP standards and their corresponding endogenous signals in Sh-control and knockdown (Sh#1 + 2) MCF7 cells. The x-axis shows retention time (minutes), and the *y*-axis indicates signal intensity (counts). ALKBH, AlkB homolog.

Since ALKBH6 protein could demethylate 7me-GMP and 1me-AMP *in vitro*, we decided to see the effect of ALKBH depletion on the intracellular level of 7me-GMP and 1me-AMP. We downregulated ALKBH6 protein in MCF7 cells by expressing two shRNAs (Fig. S6), one of which (Sh-ALKBH6#1) was published earlier (23). Quantitative evaluation of mRNA level by reverse transcriptase polymerase chain reaction revealed that expression of both the shRNAs substantially silenced ALKBH6 expression (Fig. 7*A*). Silencing was further confirmed in cotransfection experiments with GFP-ALKBH6. We found that the ALKBH6-specific shRNA silenced the target (Fig. 7*B*). As shRNAs were found to be efficient in silencing ALKBH6, we next determined the effect on intracellular N-methylated nucleotides level in ALKBH6 knockdown MCF7 cells. For this, nucleotide pools were extracted from the cells and using a 7me-G–specific competitive ELISA and 7me-GMP standard (Fig. S13), 7me-GMP concentration was found to increase from 9.06 (±0.5) ng/ml in control MCF7 cells to 12.78 (±1.77) ng/ml in ALKBH6-knockdown cells. The samples were further analyzed by electrospray ionisation-quadrupole time-of-flight (ESI-Q-TOF) mass spectrometry to determine the effect of ALKBH6 knockdown on intracellular 7me-GMP and 1me-AMP levels. LC/MS experiment showed an increase of 7me-GMP and 1me-AMP levels compared to control when ALKBH6 was silenced (Fig. 7, *D*–*G*). Overall, our mass spectrometric measurement supported our hypothesis that ALKBH6 might play an important role in the demethylation of cellular N-methylated nucleotide monophosphate pool.

**Figure 8:**
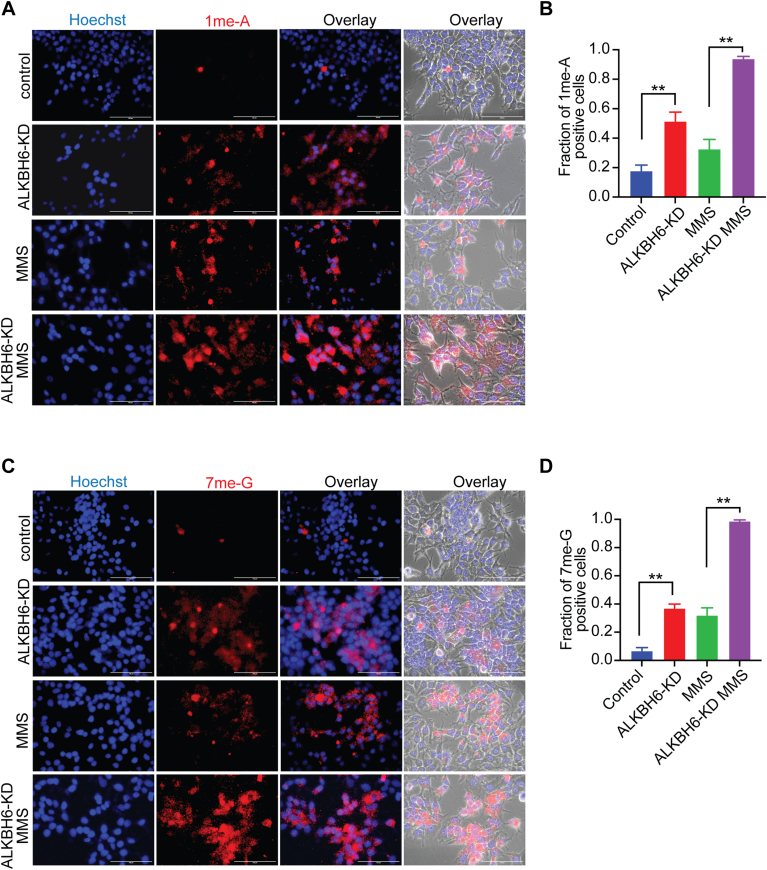
**Accumulation of 1me-AMP and 7me-GMP in the absence of ALKBH6.***A* and *C*, relative 1me-AMP and 7me-GMP levels (*red*) were determined by immunofluorescence using the antibody against the lesions in control MCF7 cells or in the cells depleted in ALKBH6. MCF7 cells were vector transfected (control) or stably transfected with plasmids encoding Sh #1 and #2 (ALKBH6-KD). For MMS treatment, MCF7 and ALKBH6-KD cells were treated with MMS (200 μM) for 24 h followed by recovery (24 h) in the absence of MMS. The permeabilized cells were stained using primary antibodies against 1me-A (rabbit) or 7meG (rabbit) (1: 250 dilution) and secondary antibody Alexa-Fluor 633 goat anti-rabbit (1:1000 dilution). Nuclei were stained using Hoechst 33258 (*blue*). The scale bar represents 100 μm. *B* and *D*, the fraction of 1me-AMP and 7me-GMP positive cells in (*A*) and (*C*). More than 500 cells were counted for each of the conditions. All data represent mean ± SE (error bars) from three biologically independent experiments (n = 3) each having 3 replicates (∗∗, *p* < 0.01). ALKBH, AlkB homolog; MMS, methyl methanesulfonate.

Since ALKBH6 is capable of oxidative demethylation of nucleotides, cells depleted for ALKBH6 should be affected in their capacity to repair methylated nucleotides. To test this hypothesis, we set to detect 7me-G and 1me-A levels by immunofluorescence using specific antibodies against 7me-G and 1me-A. The ability of these antibodies to detect cellular 7meG and 1meA was verified first by immunofluorescence analysis. These adducts were generated by treating the cells with methylating agent MMS and immunofluorescence experiment was performed with the antibodies against 7me-G and 1me-A (Fig. S14). As these antibodies were capable of detecting methyl DNA adducts in MMS-treated cells, we set to use them for detection of methylated nucleotides in normal and ALKBH6-depleted cells. While very low levels of 7me-G and 1me-A were observed in mock-transfected (control) cells, higher levels of 1me-A and 7me-G were detected in ALKBH6 knockdown cells (Fig. 8, *A* and *B*). To further enhance the basal levels of methylated nucleotides, both control MCF7 and ALKBH6-depleted MCF7 cells were treated with MMS followed by a recovery. Following treatment and recovery, the levels of 1meA and 7meG were significantly higher in ALKBH6 knockdown cells than control MCF7 cells (Fig. 8, *A*–*D*). Together, these results support our *in vitro* results and suggest that ALKBH6 is involved in the *in vivo* repair of 7me-GMP and 1me-AMP.

## Discussion

Cellular N-methylated nucleotides primarily arise from the degradation of modified RNA. As there were no known enzymes involved in the demethylation of N-methylated nucleotides and there were no known substrates for ALKBH6, an ALKBH family of demethylase, we questioned if ALKBH6 could be a demethylase for the nucleotides. We adopted three different assays, each with unique strengths, to address this question. The FDH enzyme-linked indirect assay allowed real-time enzyme activity measurement and steady-state kinetics analysis but lacked direct detection of substrate depletion or product formation. HPLC-based analysis was used to monitor substrate and product levels but it was limited to end-point analysis and required standards. High-resolution mass spectrometry offered ultrasensitive detection of nucleosides, nucleotides, and their derivatives and modified analogs and they were used for the final validation. Strikingly the favored substrate was found to be methylated mononucleotide including 7me-GMP/7me-dGMP and 1me-AMP/1me-dAMP. Maciejewska *et al.* proposed that the preference of AlkB family enzymes toward positively charged lesions is likely due to the presence of negatively charged aspartate residue in the active site (45). Evidently, ALKBH6 substrates examined here, including 7me-GMP, 1me-AMP, and 3me-CMP, the leaving methyl group is attached to a positively charged tetravalent nitrogen (Fig. 6*F*). Thus, ALKBH6 might use a similar mechanism to demethylate different substrates like other AlkB family members. Our *in vitro* findings were supported by *in vivo* experiments suggesting that 7me-GMP and 1me-AMP could be endogenous target of ALKBH6.

There are evidence in the literature suggesting that oxidized or alkylated nucleotides can be incorporated into DNA and RNA, potentially contributing to replication or transcription stress. For example, in the absence of nucleotide pool sanitization by enzymes such as MTH1 (46), oxidatively damaged guanine nucleotides (*e.g.*, 8-oxo-dGTP) can be incorporated into DNA, leading to genomic instability and cytotoxicity (47). RNA polymerases might also be vulnerable to nucleotide mis-incorporation during RNA synthesis (20, 21, 48). Thus, demethylation of the nucleotide pool by ALKBH6 might be crucial for safeguarding the cellular DNA and RNA metabolism.

What could be the cellular source of N-methylated nucleotides, the key substrates of ALKBH6 identified here? Emerging evidence suggests that they are mainly added by the specific methyl-transferase enzymes. For example, 7me-G methylation within mRNA, tRNA, and miRNAs are catalyzed by METTL1/WDR4 (4, 5, 6); 3me-C methylation in tRNA is catalyzed by METTL6 and METTL2A (7, 8, 9); 1me-A methylation in tRNA, rRNA, and mRNA of eukaryotic cells (10) are added by six different methyl transferases (49). These internally located 7me-G, 1me-A, and 3me-C adducts in mRNA are released as 7me-GMP, 1me-AMP, and 3me-CMP, respectively, during mRNA degradation (50). Any exposure to exogenous methylating agents would lead to excess level of these adducts and could likely activate quality control processes resulting in the accumulation of N-methylated nucleotides (51). 7me-GMP can also arise from mRNA 7me-GpppG cap. Processing of mRNA by decapping enzyme (Dcp1/2) releases it as 7me-GDP. The histidine triad family of pyrophosphatase Aph1/FHIT catalyze hydrolysis of mRNA cap to produce 7me-GMP, whereas scavenger decapping enzyme DcpS produce 7me-GMP from 7me-GTP (52, 53).

## Experimental procedures

### Purification of recombinant proteins

ALKBH2 and ALKBH3 were cloned as described before (17, 32). His-tag AlkBH6 was a generous gift from Prof Timothy O’Connor, City of Hope, Duarte Cancer Centre. ALKBH2 mutant (H171A, D173A), ALKBH3 mutant (H191A, D193A, H257A), ALKBH6 mutant (H114A, D116A, H182A) and single catalytic mutant ALKBH6 (H182A) were generated by site-directed mutagenesis. Please see the supplementary method section Table S1 and Fig. S2 for the details. WT and mutant recombinant proteins were purified using standard Ni-affinity followed by gel-filtration chromatography and stored in 20 mM Tris–HCl, pH 8.0, 150 mM KCl, glycerol 5% (v/v) as reported earlier (17, 32).

### Detection of uncoupled succinate production

The uncoupled succinate production reaction was set up in two steps as described before (54). In the initial step, WT ALKBH6 (5–20 μM) was incubated in dioxygenase reaction buffer (20 mM Hepes, pH 8.0, 0.2 mM 2OG, 2 mM ascorbate, 5 μM Fe(NH_4_)_2_(SO_4_)_2_) for 1 h at 37 °C to form succinate. Then the reaction mixture (50 μl) was mixed with recombinant sucCD (1 μM) in SucCD reaction buffer (2 mM MES, pH 6.5, 5 mM ATP 3.258 mM CoA, 50 mM MgCl_2_) and further incubated for 30 min at 37 °C. Finally, molybdate solution was added for colorimetric detection and the succinate concentration was detected from the standard curve. For inhibition, CoCl_2_ (20 μM) was used. The results were plotted and analyzed using GraphPad Prism (www.graphpad.com).

### Demethylation assay

NMPs including 1meAMP (Jena Bioscience, NU-1029-1,) 7meGMP (Jena Bioscience, NU-1135S), 6me-AMP (Sigma, M2780) were purchased. N-methylated deoxy-NMPs were prepared by methylating chemically synthesized 30-mer oligonucleotide containing either dA, dG, dC, or dT by MMS (5%, v/v) (0.59 M) MMS (Sigma, 129925) and purified as described before (17). The oligonucleotides were digested with nuclease P1 (NEB) at 37 °C for 4 h to produce deoxy-NMPs. After heat inactivating the nuclease, dNMPs were purified by HPLC and used for the demethylation reaction. N-methylated NMPs were purified similarly, using RNA as the starting material. For the demethylation reaction, ALKBH6 (1 μM) and me-dNMPs (1 mM) were mixed in the dioxygenase reaction buffer and incubated at 37 °C for 30 min. Formaldehyde release was detected by FDH-coupled DNA repair assay as described before (32, 33). Briefly, 1 mM NAD+ and 0.01 U FDH were added to the dioxygenase reaction buffer containing methylated nucleotide and ALKBH6. NADH generated was detected by the characteristic absorption peak (340 nm) using a 96-well multimode reader (Synergy, Biotek Instrument). Formaldehyde production was quantified from the standard curve and the initial velocities were determined using various substrate concentrations. The results were plotted and analyzed using GraphPad Prism.

### Demethylation assay by HPLC analysis

Following ALKBH6-mediated demethylation, NMPs and dNMPs were analyzed in an HPLC system (Shimadzu) equipped with a reversed-phase Shim-pack GIST C18 5 μm separation column (250 × 4.6 mm). For majority of the nucleotides including 1me-AMP, 1me-dAMP, 7me-dGMP, 6me-AMP, 7me-GMP, 3me-dCMP, dAMP, dCMP AMP, GMP, the column was equilibrated with mobile phase A (50 mM ammonium acetate) and mobile phase B (50 mM ammonium acetate, 50% of acetonitrile and 0.1% trifluoroacetic acid. For dGMP, different mobile phase A (50 mM ammonium acetate, 3% methanol, pH-5.0) and mobile phase B (methanol) were used. The peaks were identified based on the elution time of the standards (Fig. S9) purchased from Sigma and further analyzed using LabSolutions software (www.shimadzu.com) and analyzed by mass spectrometry.

### ESI-Q-TOF mass spectrometry

Analyte separation was performed using an Agilent 1290 Infinity II system equipped with a Zorbax SB-C18 reversed-phase column (2.1 × 100 mm, 1.8 μm). The mobile phase comprised water with 10 mM ammonium acetate (solvent A) and methanol (solvent B), employing a binary gradient (20% B) elution over 5 min at 0.250 ml/min flow rate. Nucleotide samples derived from the MCF7 cell line were directly injected into the ultra-high performance liquid chromatography-MS system with a binary gradient (20% B) over 2 min. The injection volume was 2 μl with a 3-s needle wash. The column temperature was maintained at 40 °C, and UV absorption spectra were monitored using a DAD with a scan range from 190 nm to 400 nm. Mass spectrometric detection was performed on a high-resolution liquid chromatography system coupled with a quadrupole time-of-flight mass spectrometry (Agilent 6550 iFunnel ESI-Q-TOF equipped with a dual AJS electrospray ionization source) in positive ion mode with a scan range from 100 to 750 *m/z*. Source and interface parameters were optimized for efficient ionization, with data acquisition at 1 spectrum per second. Instrument performance was evaluated using six replicate injections of sulfadimethoxine qualification standard. System suitability tests included RT, mass accuracy, and reproducibility assessment (Table S2). Data processing was carried out using the Agilent MassHunter Acquisition and Qualitative Analysis software (www.agilent.com) for mass spectra generation and visualization, incorporating +extracted ion chromatogram, total ion chromatogram, DAD chromatogram, and tandem mass spectrometry fragmentation patterns. Target spectra were identified using the Find by Molecular Formula feature in the compound view workflow.

### Intrinsic tryptophan fluorescence emission of ALKBH6

All tryptophan fluorescence spectroscopy experiments were performed using a multimode plate reader (PerkinElmer). The excitation and emission wavelengths were fixed at 280 nm and 356 nm, respectively. To measure ALKBH6–ligand interactions, recombinant ALKBH6 (500 nM), and increasing concentrations of ligand (0–80 μM) were equilibrated in binding buffer (50 mM Hepes, pH 7.6, 300 mM KCl) for 1 h. For Stern–Volmer quenching analysis, the *F*_*0*_*/F = 1 + K*_*SV*_*(L)* formula was used, where, *K*_*SV*_ is the Stern–Volmer constant, *L* is the concentration of the quencher, *F*_*0*_ and *F* represent the steady-state fluorescence intensities in the absence and presence of quencher, respectively. All the data were calculated with best-fit parameter values and 95% confidence intervals in GraphPad Prism software.

### Thermal shift analysis

All the experiments were performed in Eco Real-Time PCR system as described before (55). Purified recombinant His-tag ALKBH6 or ALKBH6-Y120A (5 μM) was incubated with varying concentrations (0–50 μM) of 7methyl-GMP in binding buffer (50 mM Hepes pH7.6, and 75 mM KCl). SYPRO Orange was added to the reaction (20 μl) and melting derivatives (-*d*RFU/*d*T) of fluorescence emission were determined by measuring the fluorescence with temperature increment of 0.1 °C from 40 °C to 100 °C. The ΔT_m_ was calculated by subtracting T_m_ of ALKBH6 from T_m_ of ALKBH6 with 7methyl-GMP. The changes of Tm (ΔT_m_) were plotted against increasing concentrations of 7methyl-GMP and dissociation constant was determined by using one site specific binding formula of GraphPad Prism software.

### Expression constructs

The GFP-tagged ALKBH6 expression plasmids were constructed by cutting the PCR-amplified WT ALKBH6 with XhoI and EcoRI, and then cloned in-frame and upstream of the sequence encoding the GFP pEGFP-N1 (Clontech). ALKBH6 shRNA constructs were generated using reported sequences (shALKBH6#1 5′ AGGAGTATTTGCTTCGACA 3′, shALKBH6#2 5′ GCACCACCTGTAATCTACTAT 3′) and cloned into the pSilencer 2.1-U6 puro shRNA vector. All the shRNA constructs were confirmed by sequencing (Fig. S6). For negative control, pSilencer 2.1-U6 puro vector was used.

### Cell culture, RNAi, and transfection

MCF7 cells were maintained in Dulbecco's modified Eagle's medium (DMEM) with 10% fetal bovine serum at 37 °C in a humidified air containing 5% CO_2_. Stable ALKBH6 knockdown cell line was generated by inserting the target sequence for ALKBH6 or the scramble control into the pSilencer2.puro (pSil) vector (Ambion) to form short hairpin RNAs for RNAi. For plasmid transfection in MCF7 cells, cells were suspended in DMEM without fetal bovine serum or antibiotics at a concentration of 1 × 10^7^ cells/ml. A volume of 0.1 ml was transferred to a sterile electroporation cuvette (Bio-Rad Gene Pulser cuvette) and kept at room temperature for 5 min in the presence of 15 μg plasmid. Electroporation was performed using the Gene Pulser Xcell System (Bio-Rad) with 220 V/cm, 950 μF capacitance, and infinite resistance. After receiving the electric pulse, cells were transferred to culture flasks and incubated with complete DMEM. The gene knockdown was confirmed by reverse transcriptase polymerase chain reaction (Supplementary method and Table S2). Stable ALKBH6 knockdown cell lines were prepared by transfecting MCF-7 cells with shRNA-ALKBH6. Postelectroporation, cells were selected with 0.5 μg/ml puromycin for 3 weeks and ALKBH6 knockdown was confirmed by RT-qPCR and mRNA expression analysis. For evaluation of effect of MMS, cells were treated with MMS (200 μM) for 24 h followed by recovery (24 h) in the absence of MMS. For microscopic analysis, GFP-ALKBH6 construct were electroporated in MCF7 cells and grown on chamber slides for 48 h. Cells were then washed with PBS and clear DMEM w/o Phenol red (Himedia) media were added. Nuclear DNA was stained using Hoechst 33258 (Sigma).

### Extraction of intracellular nucleotides

Cellular nucleotides were isolated using the method reported earlier (56), with some modifications. For the experiment, 4 × 10^6^ cells were removed by scrapping, washed five times with Hanks' balanced salt solution, and resuspended in an ice-cold extraction buffer, comprising acetonitrile, methanol, and water in a ratio of 40:40:20 (v/v/v) or 20:40:40 (v/v/v), followed by incubation at 4 °C for 15 min. Subsequently, the lysates were sonicated and centrifuged at 10,000 *g* for 10 min at 4 °C to remove the cell debris. The supernatant is then transferred to an Amicon Ultra Centrifugal Filter, 3 kDa (Millipore, UFC8003) and centrifuged at 4400 rpm, 30 min to remove macromolecules. The filtrate was collected and evaporated to dryness using a vacuum evaporator at 45 °C. The dried fraction was resuspended in ultrapure water (50 μl) and subsequently used for HPLC, ELISA, and ESI-Q-TOF mass spectrometry.

### Immunofluorescence imaging of 1meA and 7meG

MCF-7 cells were electroporated (5 × 10^4^ cells per well) with or without ALKBH6 shRNA constructs and incubated for 48 h on 24-well plates. Cells were washed with Hanks' balanced salt solution buffer and fixed using 4% formaldehyde. After fixation, cells were treated with 1.5 M HCl for 20 min, followed by neutralization with sodium borate (pH 8.5) for 2 min. Cells were permeabilized with 0.1% Triton X-100 for 10 min. After blocking with bovine serum albumin (1% v/v) cells were incubated with primary antibodies against 1me-A (rabbit, Abcam, ab208196) or 7meG (rabbit, Abcam, ab300740) at 1: 250 dilution and secondary antibody Alexa-Fluor 633 goat anti-rabbit at 1:1000 dilution. Nuclear DNA was stained using Hoechst 33258 (Sigma). Microscopy was performed with a fluorescence microscope (EVOS FL Auto, Thermo Fisher Scientific) under 40× magnification. The images were processed using ImageJ (imagej.net).

## Data availability

This study includes no data deposited in external repositories. All the data used in the experiment are presented. Additional data are presented in the supplementary section. Any other data will be made available on request.

## Supporting information

This article contains supporting information.

## Conflict of interest

The authors declare that they have no conflicts of interest with the contents of this article.
