# Supplementary material for: Identification of ALKBH6 as a nucleotide demethylase with a distinct substrate preference
Source: J Biol Chem. 2025 Aug 28;301(10):110638. doi: 10.1016/j.jbc.2025.110638 (PMC12494543; doi:10.1016/j.jbc.2025.110638)
Supplement: Supporting Figures and Tables [file mmc1.docx]

**SUPPLEMENTARY INFORMATION**

**Identification of ALKBH6 as a nucleotide demethylase with a distinct substrate preference**

Susmita Das^ψ^, Sourbh Rankawat^ψ^, Unnikrishnan P Shaji^ψ^, Nikhil Tuti, Nafeesa Shahnaz, Sandipan Ray*, Roy Anindya^,^*

**SUPPLEMENTARY METHODS**

**Site directed mutagenesis:** To generate ALKBH2 catalytic site mutant, amino acids His 171, Asp 173 and His 236 were mutated to Ala. To generate ALKBH6 catalytic site mutant amino acids His 114, Asp 116 and His 182 were mutated to Ala. Additionally, Y120 and E115 of ALKBH6 were also mutated to Ala to check the binding residue of ALKBH6. All the mutations were carried out using site-directed mutagenesis using specific primers (**Supplementary Table S1**) and using pET28a-ALKBH2 and pET28a-ALKBH6 clones as template. The strategy to generate mutations involved generation of a megaprimer containing mutation. For this, forward and reverse primers were used for PCR amplification using conventional PCR cycle using Phusion-Hifi DNA polymerase (Thermo). Then the PCR product (mega-primer) was cleaned up using PCR clean-up kit and used for amplification using Phusion Hifi DNA polymerase. PCR condition for the whole-plasmid amplification (pET28a-ALKBH2 or pET28a-ALKBH6) involved initial denaturation at 98˚C for 10 s, denaturation for 10 s, primer annealing at 57˚C for 30 s, primer extension at 72˚C for 7 min and final extension at 72˚C for 10 min. Following 18 cycles of whole-plasmid amplification, the PCR product was digested with DpnI and transformed into *E. coli* DH5α.

**Reverse-transcriptase quantitative PCR:** Total RNA was purified with the RNeasy kit (Qiagen) and treated with the RNase-Free DNase (Qiagen) to remove the residual DNA. 1.6 μg of RNA was reverse transcribed using the first strand cDNA synthesis reverse transcription kit (Thermo-scientific, K1622) and oligo (dT) primers. Quantitative PCR was performed using cDNA (160 ng) sample obtained from three independent experiments, with triplicate sample for each experiment, using the Universal SYBR Green Supermix Kit (Bio-Rad,1725270). The sequences of all primers used are listed in the **Supplementary Table S2.** The housekeeping gene β-ACTIN were used as the internal reference genes. The fold change in expression of the target gene relative to the reference genes was assessed. The RT-qPCR data were presented as the fold-change in gene expression normalized to the reference genes and relative to the control.

**Molecular docking:** Crystal structure of Hollo enzyme ALKBH6 (PDB ID: 7VJV) was retrieved from RCSB Protein Data Bank. For receptor preparation, water molecules were removed, polar hydrogen and Kollman charge were added using Auto Dock Tools (ADT). The structure of the protein was saved in PDBQT format for further analysis. Structure of 7meGMP and 1meAMP was drawn in Chemsketch and saved as mol2 Format. Structure energy minimisation was done with AVOGADRO software to achieve stable conformation. Ligands were auto-optimized (addition of Gasteiger charges and detection of rotatable bonds) and exported in PDBQT format using Autodock MGL Tools. The grid centre for docking was set as X= 13.202, Y = -4.161, and Z= -0.305 with dimensions of the grid box 80×64×64 Å. Throughout the docking, ALKBH2 was kept rigid while the ligand were kept flexible. Docking was run on the Lamarckian genetic algorithm. The number of docking runs was set to 100 for each inhibitor, all other parameters were left at their default settings. Interaction analysis was done using Bio via Discovery studio and PLIP web server. Images where prepared with PyMol.

**Quantification of 7me-GMP concentration by ELISA:** For the ELISA based detection and quantification of 7me-GMP, competitive ELISA Kit (RayBiotech, EIA-m7G) was used. Initially standard sigmoidal graph was prepared using 7me-GMP standard with a concentration range from 200 μg/mL to 0.002 ng/mL as per the instruction of the manufacturer. Samples (50 μL) containing cellular nucleotide pools (in duplicates) were prepared from three biological replicates and added to the wells of 8 well-strips. The nucleotide pool obtained from ALKBH6 knockdown MCF7 cell line was compared with the control MCF7 cell line. ELISA followed by HRP-based detection was carried out exactly as suggested by the manufacturer. The readings were obtained from a plate reader Synergy (BioTek instrument) set to measure absorbance at 450 nm. Finally, the ratios of the ELISA absorbance values of antibody-binding response in the sample (B) to the blank (B_0_) were plotted (Y axis) against the log_10_ of 7me-GMP concentrations (X axis) and fitted using GraphPad Prism software. The interpolated values were converted to antilog for obtaining the exact concentration of 7me-GMP in each sample of control and knockdown cell lines.

**SUPPLEMENTARY TABLES**

**Table S1.** Sequence of primers used in the study for site-directed mutagenesis

| Primer name | Primer sequence |
| --- | --- |
| H171A D173A (Forward) | GTGACCACATCGGGGAGGCCCGAGCTGATGAAAGAGAACTGGCCC |
| H236A (Reverse) | CCTTCTTTCTCACGGGAAGACTGGCGTACCAGTGCGTGTTGGTC |
| H114A D116A (Forward) | GGAGGGCATCATGCCCGCCGAGGCCGGACCACTGTACTAC |
| H182A (Reverse) | CGCGGGCGGCGGCGATGCCGGCGAGAAGACGCGTGTAGGC |
| Y120A (Forward) | CCCACGAGGACGGACCACTGGCCTACCCGACTGTCAGCACCA |
| Y120A (Reverse) | CGCGGGCGGCGGCGATGCCGTGGAGAAGACGCGTGTAGGC |
| E115A (Forward) | GGAGGGCATCATGCCCCACGCGGACGGACCACTGTACTAC |
| E115A (Reverse) | CGCGGGCGGCGGCGATGCCGTGGAGAAGACGCGTGTAGGC |

**Table S2.** Sequence of primers used in the study for quantitative RT-PCR

| Primer name | Primer sequence |
| --- | --- |
| ALKBH6 (Forward) | GTCCTCGTGAACCAGTATCTGC |
| ALKBH6 (Reverse) | TCGTAGAAGTCCAGCACGGTGT |
| Beta-actin (Forward) | ATCCACGAAACTACCTTCAA |
| Beta-actin (Reverse) | ATCCACACGGAGTACTTGC |

**Table S3.** ALKBH6 amino acid residues involved in the interaction with its ligands

| **Ligand: 7me-GMP** | | **Ligand: 1me-AMP** | |
| --- | --- | --- | --- |
| **Interacting residues** | **Type of interaction** | **Interacting residues** | **Type of interaction** |
| Arg56 | Hydrogen bond | Arg56 | Hydrogen bond |
| Lue58 | Van der Waals | Lue58 | Van der Waals |
| Asn60 | Hydrogen bond | Asn60 | Hydrogen bond |
| Pro65 | Carbon-Hydrogen bond | Pro65 | Van der Waals |
| His99 | Carbon-Hydrogen bond | His99 | Carbon-Hydrogen bond |
| Lue101 | Van der Waals | Lue101 | Van der Waals |
| Ile111 | Van der Waals | Ile111 | Pi-Alkyl |
| His114 | Pi-pi stacked | Met112 | Carbon-Hydrogen bond |
| Glu115 | Hydrogen bond | pro113 | Van der Waals |
| Asp116 | Hydrogen bond | His114 | pi-pi stacked |
| Gly117 | Van der Waals | Asp116 | Hydrogen bond |
| Tyr120 | Hydrogen bond | Tyr120 | Hydrogen bond |
| Arg224 | Hydrogen bond | Arg224 | Hydrogen bond |

**Table S4.** System Suitability Test (SST) using qualification standard Sulfadimethoxine

| **S. No.** | **Injection** | **RT DAD** | **RT MS** | **Precursor ion mass** | **Daughter ion mass** | **Peak area (DAD)** | **Peak area (MS)** |
| --- | --- | --- | --- | --- | --- | --- | --- |
| 1 | 1.00 | 0.26 | 0.36 | 311.08 | 156.08 | 30.00 | 31733030.00 |
| 2 | 2.00 | 0.26 | 0.36 | 311.08 | 156.08 | 30.00 | 31751554.00 |
| 3 | 3.00 | 0.27 | 0.36 | 311.08 | 156.08 | 30.00 | 32479847.00 |
| 4 | 4.00 | 0.27 | 0.36 | 311.08 | 156.08 | 31.00 | 30291993.00 |
| 5 | 5.00 | 0.26 | 0.36 | 311.08 | 156.08 | 30.00 | 31316682.00 |
| 6 | 6.00 | 0.27 | 0.37 | 311.08 | 156.08 | 31.00 | 34414682.00 |
| **Mean** | | 0.26 | 0.36 | 311.08 | 156.08 | 30.33 | 31997964.67 |
| **Standard deviation** | | 0.00 | 0.01 | 0.00 | 0.00 | 0.52 | 1384061.22 |
| **% RSD** | | 0.62 | 1.55 | 0.00 | 0.00 | 1.70 | 4.33 |

**SUPPLEMENTARY FIGURES**

**Figure S1. Demethylation of methylated nucleotides and formaldehyde generation by ALKBH2, ALKBH3 and ALKBH6.** Time-courses of product (formaldehyde) formation catalysed by ALKBH2, ALKBH3 and ALKBH6 in the presence of methylated-NMP. Comparative analysis of formaldehyde production by ALKBH6 is shown as reference. Further details are in Figure 1. All data represent mean ± S.E. (error bars) from five biologically independent experiments (n = 3), each having 3 replicates.

**Figure S2.** Site-directed mutagenesis and purification of recombinant ALKBH6 **(A)** Sequence analysis of wildtype ALKBH6, three catalytic site mutant (H114A, D116A and H182A) and single catalytic site mutant (H182A) **(B)** Sequence analysis of ALKBH6 binding site mutant (E115A and Y120A) **(C)** SDS-PAGE analysis of purified ALKBH6, binding site mutant ALKBH6 triple catalytic site mutant (H114A, D116A, H182A) and single catalytic site mutant ALKBH6 (K182A).­­ It should be noted that the protein loading is not equal for all proteins.


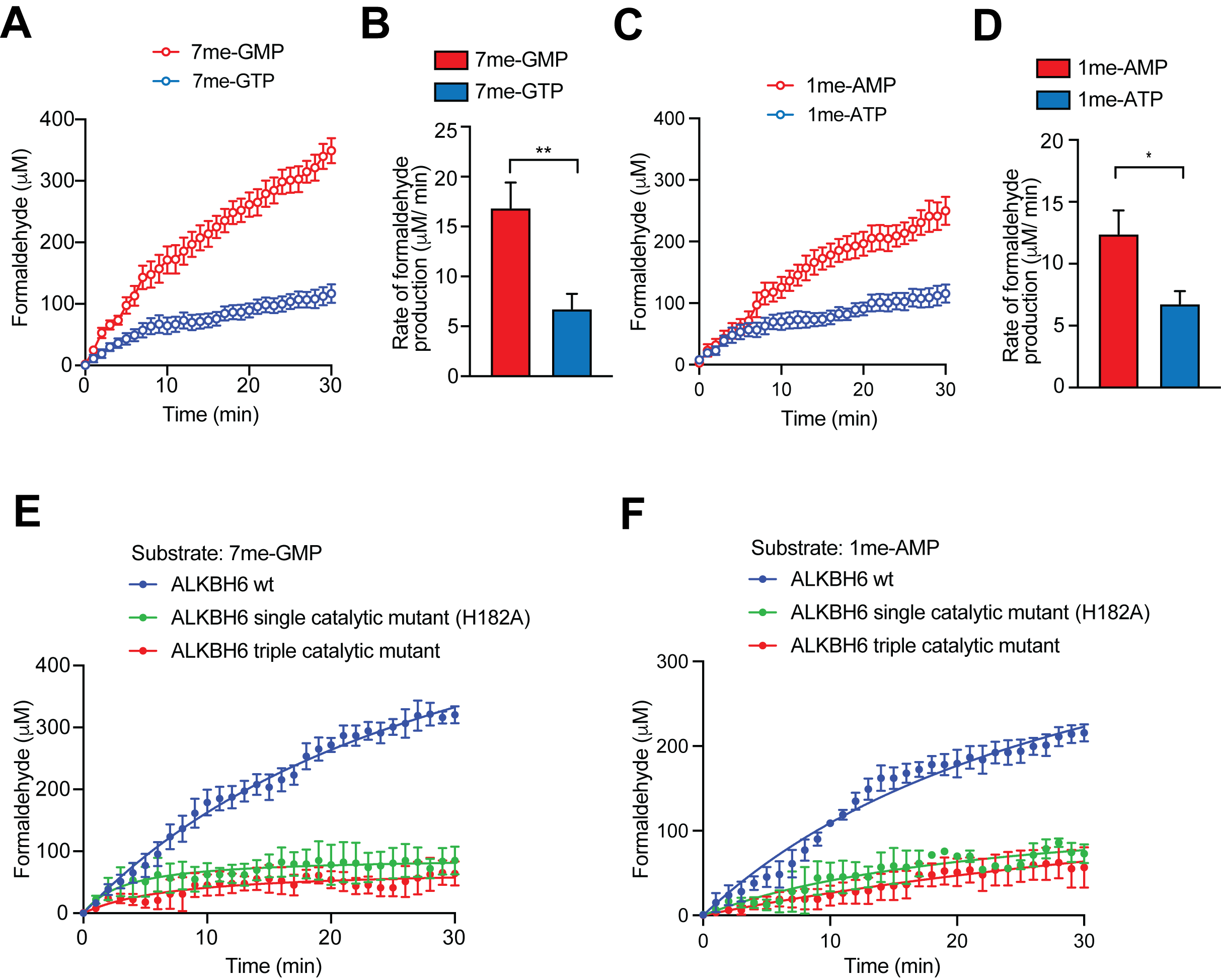


**Figure S3. Demethylation of 7me-GTP and 1me-ATP and formaldehyde generation by ALKBH6. (A)** Time courses of formaldehyde production in the presence of ALKBH6 and either 7me-GMP or 7me-GTP as substrate **(B)** Comparative analysis of rate formaldehyde production with 7me-GMP or 7me-GTP as substrate. **(C)** Time courses of formaldehyde production in the presence of ALKBH6 and either 1me-AMP or 1me-ATP as substrate **(D)** Comparative analysis of rate formaldehyde production with 1me-AMP or 1me-ATP as substrate. **(E)** Time courses of formaldehyde production in the presence of wildtype ALKBH6, triple catalytic mutant of ALKBH6 (H114A, D116A, H182A) and single catalytic mutant ALKBH6 (H182A) and 7me-GMP as substrate. **(F)** Time courses of formaldehyde production as in (E) using 1me-AMP as substrate. All data represent mean ± S.E. (error bars) from five biologically independent experiments (n = 5), each having 3 replicates. *, p < 0.05, **, p < 0.01.

**Figure S4. Binding analysis of 7me-GTP and 1me-ATP by ALKBH6. (A)** Plot of Trp fluorescence quenching by titrations with 7me-GTP; for reference 7me-GMP is shown. (**B**) Plot of Trp fluorescence quenching by titration with 1me-ATP; for reference 1me-AMP is shown. F, fluorescence in the presence of ligand; F_0_ , fluorescence in the absence of ligand. All data represent mean ± S.E. (error bars) from five biologically independent experiments (n = 5), each having 3 replicates.

**
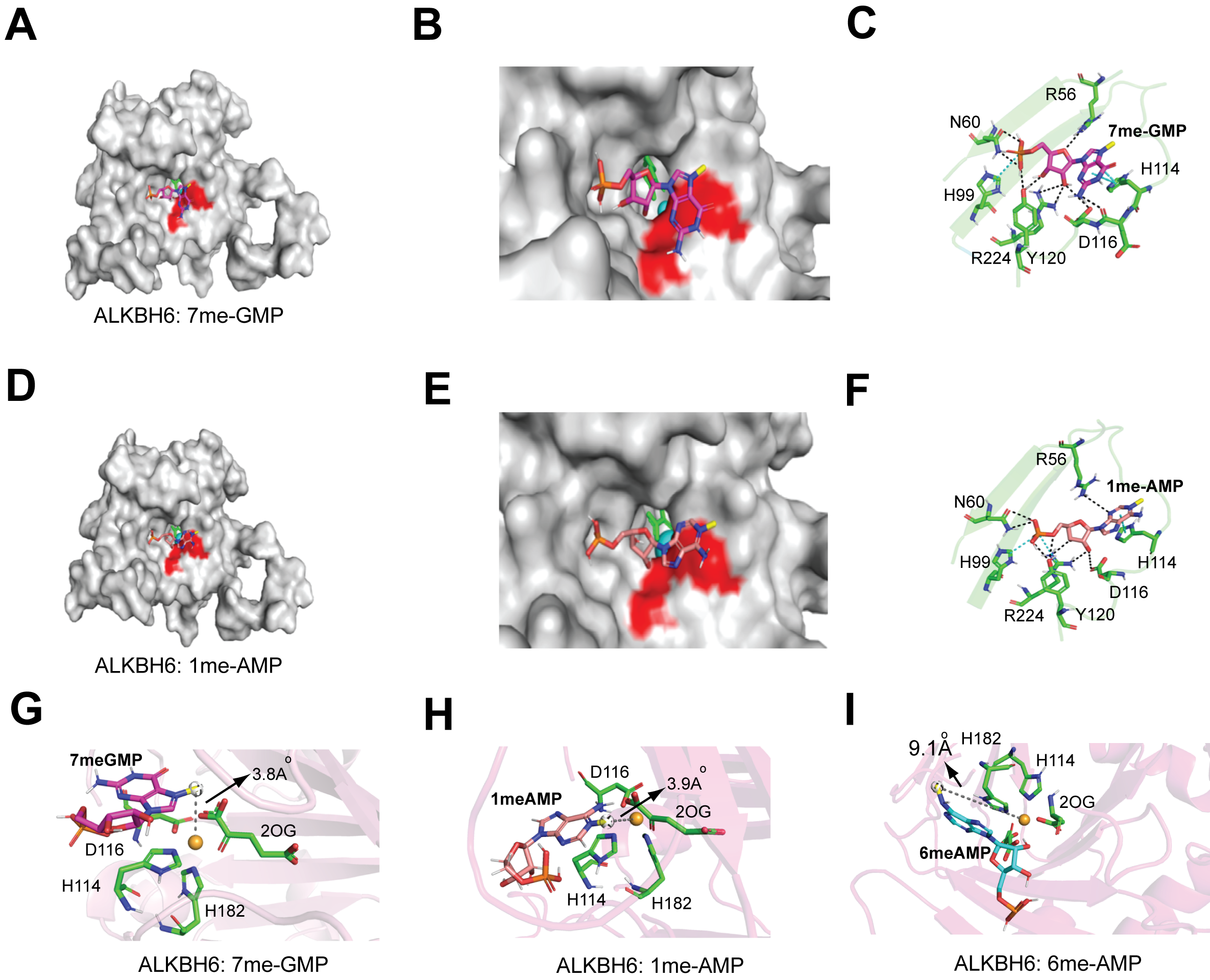
**

**Figure S5.** Molecular docking of 7meGMP and 1meAMP with human ALKBH6 (PDB: 7VJV). **(A)** The docked pose of ALKBH6-bound 7me-GMP. **(B)** Closeup view within the active site, the binding pocket of ALKBH6 (surface model) showing 7me-GMP (stick model). **(C)** Structural model showing catalytic iron, co-substrate 2-oxoglutarate (2-OG) and amino acid residues of ALKBH6 important for binding and catalysis. **(D)** The docked pose of ALKBH6-bound 1me-AMP. **(E)** Closeup view within the active site, the binding pocket of ALKBH6 (surface model) showing 1me-AMP (stick model). **(F)** Structural model showing catalytic iron, co-substrate 2-OG and amino acid residues of ALKBH6 important for binding and catalysis. (**G**) Structural model of 7meGMP docked into the active site of ALKBH6 (PDB ID: 7VJV). The docking model illustrates the binding orientation of 7meGMP within the active site of ALKBH6. Catalytic residues H114, D116, and H182 are shown. The position of the methylated guanine base () is highlighted, and the distance between leaving group carbon of the methyl group of 7meGMP and the metal ion is indicated. (**H**) Structural model of 1meAMP. The position of the methylated guanine base is highlighted. (**I**) Structural model of 6meAMP. The position of the methylated guanine base is highlighted. This spatial measurement provides insight into the lack of demethylation of by ALKBH6. (color code: ALKBH6 surface model (grey). Ribbon mode; (light pink) with 7me-GMP, 1me-AMP binding to active site (2OG, green; Mn ion, Cyan (surface model) & Golden (ribbon model); catalytic residues (His 114, Asp116, H182), Red. 7me-GMP, 1me-AMP and 6me-GMP are shown in stick model (Pink, 7me-GMP Carbon; Light pink, 1me-AMP Carbon; Light blue, 6me-AMP Carbon; Blue, Nitrogen; White, Hydrogen; Red, Oxygen; Orange, Phosphate; Yellow, Methyl group.)

**
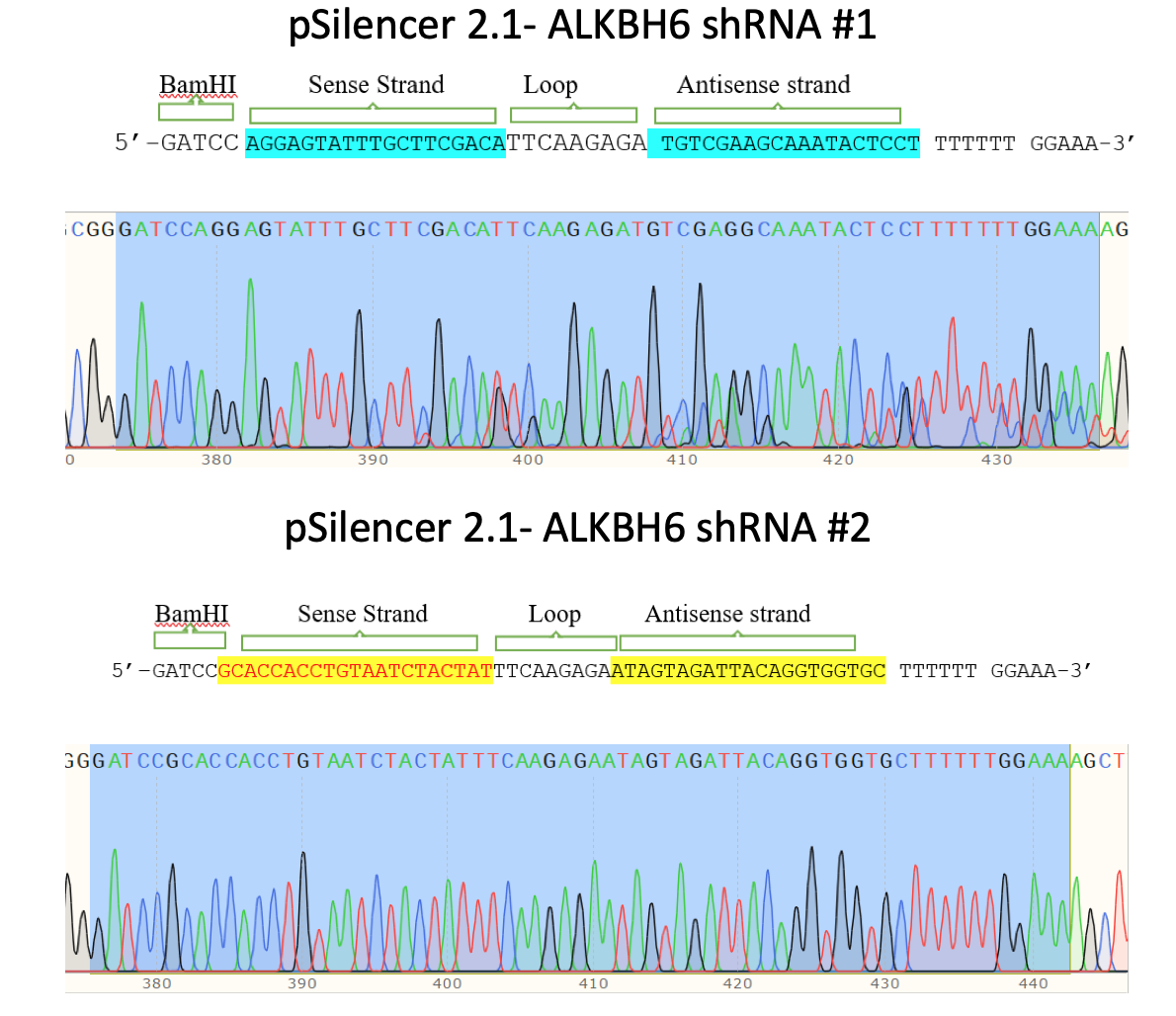
**

**Figure S6.** Sequencing of pSilencer-ALKBH6-shRNA #1 and #2. Synthetic oligonucleotides were annealed *in vitro* and ligated to pSilencer 2.1 vector cut with BamHI and HindIII. The pSilencer-ALKBH6-shRNA #1 and #2 clones were confirmed by Sanger sequencing.

**Figure S7.** Retention of a series of standard nucleotide compounds on a reverse-phase high-performance liquid chromatography (HPLC) column (Shim-pack GIST C18 5 µm column (250 x 4.6 mm) flow rate 1 ml/min).­­­ (A) 1me-AMP (3.98 min) (B) 1me-dAMP (7.76 min) (C) dAMP (5.3 min) (D) AMP (2.71 min) (E) 7me-dGMP (6.02 min) (F) dGMP (8.34 min) (G) 7me-GMP (4.77 min) (H) GMP (3.48 min) (I) 3me-dCMP (4.72 min) (J) dCMP (3.10 min).

**Figure S8: Demethylation activity of ALKBH6.** HPLC analysis were carried out (A) 1me-AMP (B) 7me-GMP using three different substrate concentration (250, 500 and 750 μM). See Figure 2 for detail.

**
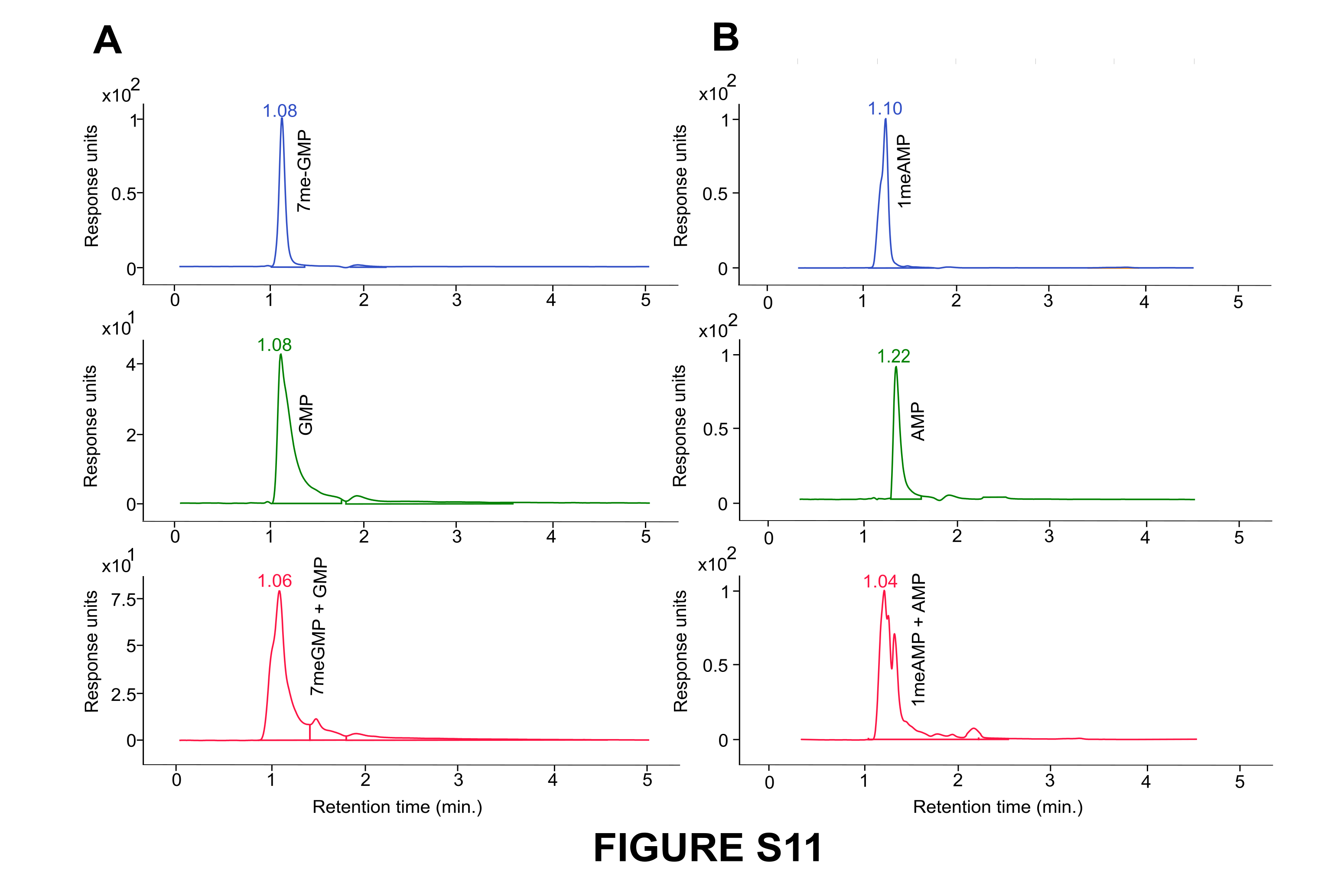
**

**Figure S9: DAD chromatograms of standard compounds and reaction mixtures.** **(A)** DAD chromatograms (260 nm) for 7meGMP (blue) and GMP (green)**,** with the reaction mixture (7meGMP + GMP) shown in red. **(B)** DAD chromatograms 1meAMP (blue) and AMP (green), with the reaction mixture (1meAMP + AMP) in red. The x-axis represents retention time (minutes), and the y-axis shows response units.

**Figure S10:** **EIC and MS spectra of nucleotide standards.** **(A)** 7-methyl-guanosine-3’-monophosphate (7-me-GMP), **(B)** guanosine-3’-monophosphate (GMP), **(C)** 1-methyl-adenosine-3’-monophosphate (1-me-AMP), (**D)** adenosine-3’-monophosphate (AMP). The x-axis represents the retention time (min) of EIC and m/z for MS spectra. The y-axis shows intensity (counts).


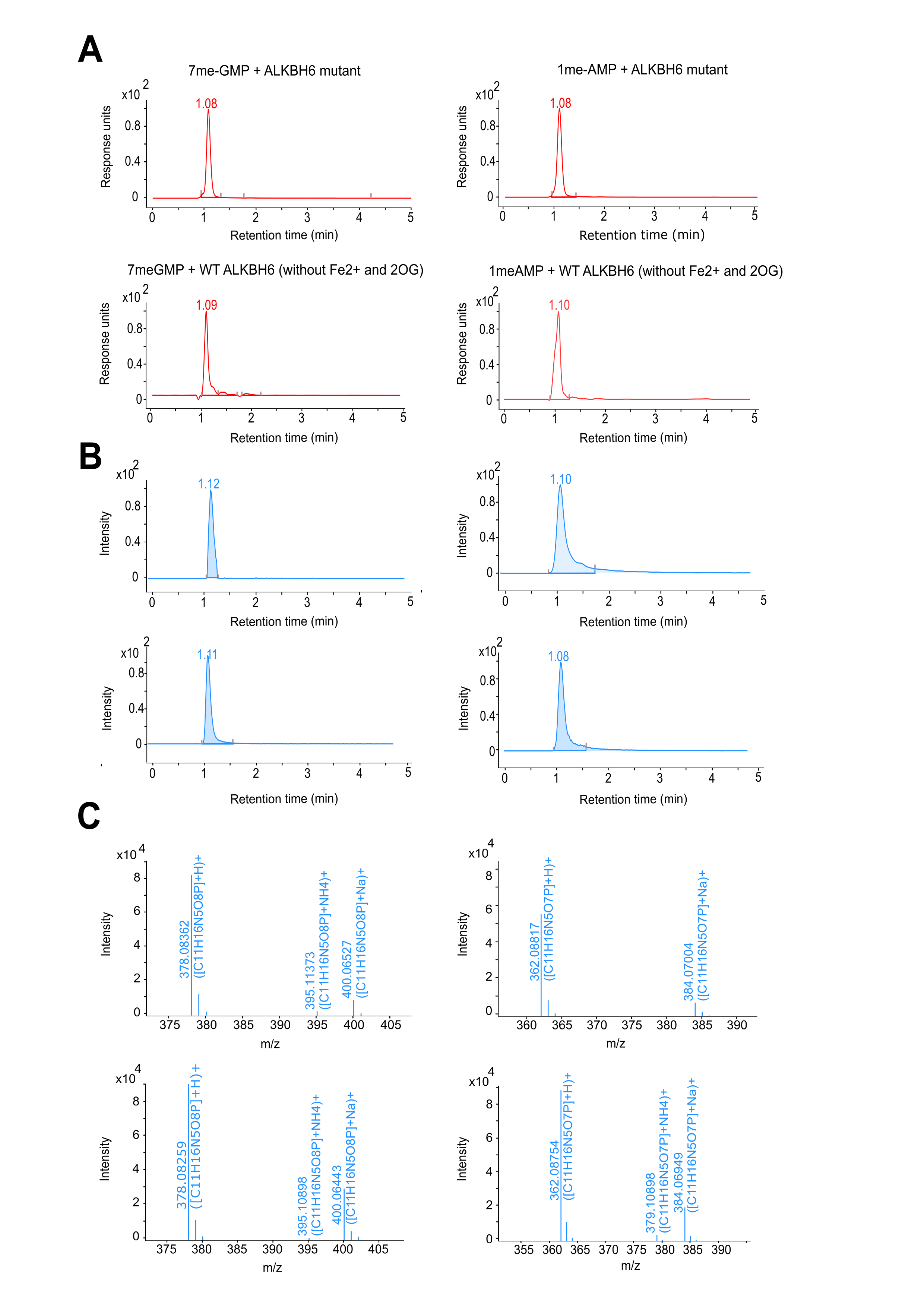


**Figure S11: LC/MS analysis of different control experimental conditions.** Negative control experiments for ALKBH6-mediated demethylation, including 7me-GMP and 1me-AMP with ALKBH6 mutants and 7meGMP and 1meAMP with wild-type ALKBH6 without Fe2+ and 2OG. **(A)** DAD signals at 260 nm (retention time vs. response units), **(B)** EICs (retention time vs. intensity), and **(C)** mass spectra (m/z vs. ion intensity).

**
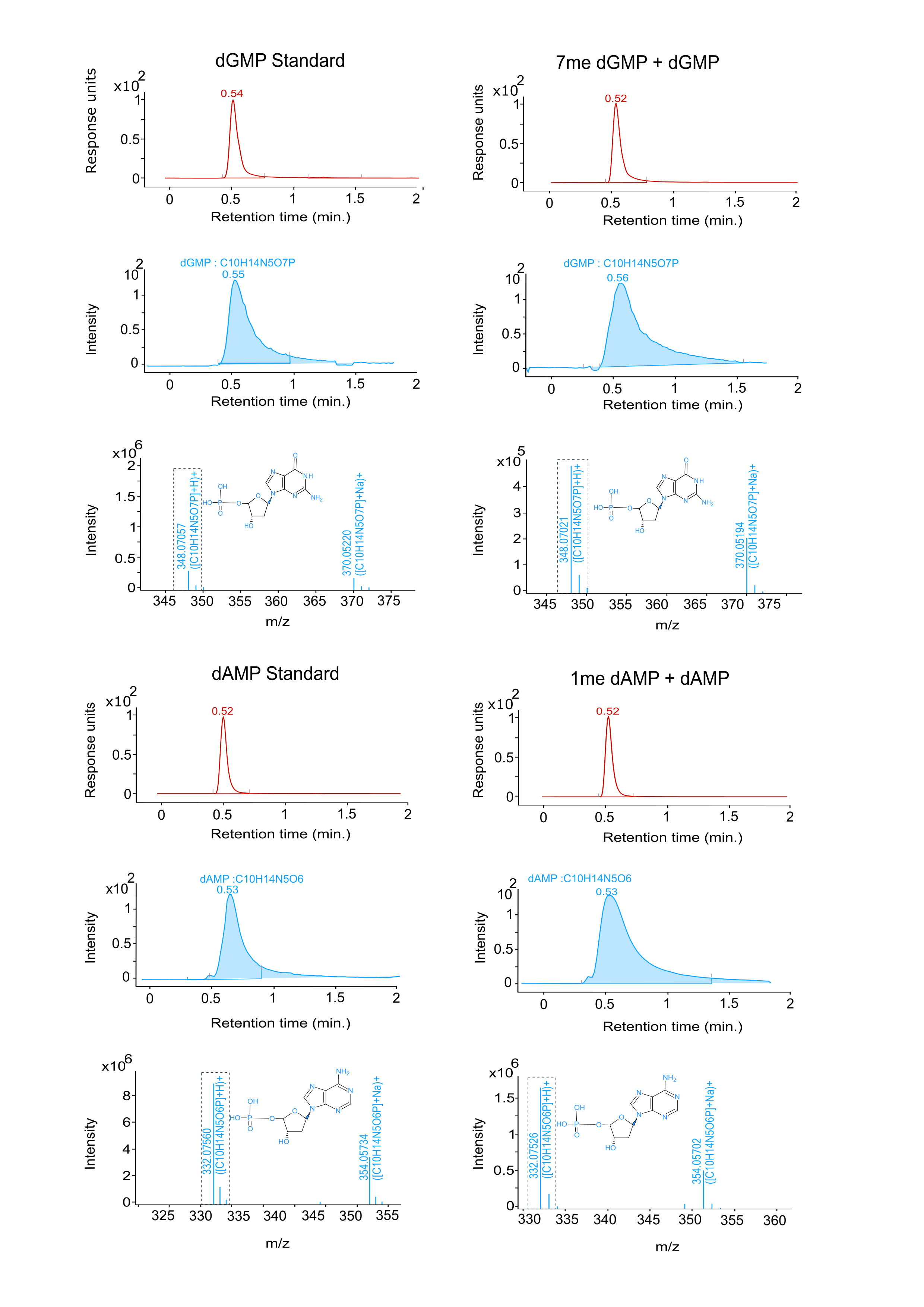
**

**Figure S12: LC/MS analysis of demethylation of deoxyribonucleotides by ALKBH6. (A)** UV absorbance (DAD, 260 nm) chromatograms (top) extracted ion chromatograms (EIC, middle) and mass spectra (bottom) for dGMP. The left panels show the standard dGMP, while the right panels show the reaction mixture containing 7me-dGMP and dGMP. **(B)** UV absorbance (DAD, 260 nm) chromatograms (top) extracted ion chromatograms (EIC, middle) and mass spectra (bottom) for dAMP. The left panels show the standard dAMP, while the right panels show the reaction mixture containing 7me-dAMP and dAMP. Separation was performed using an Agilent 1290 Infinity II system with a 2.1 × 50 mm, 1.8 µm Rapid Resolution HD column (C-18) and a mobile phase of water and 75:25 methanol-water with 5 mM ammonium formate, employing a 2-minute binary gradient at 0.6 mL/min.

**Figure S13:** Quantification of 7me-GMP concentration by ELISA. **(A)** Standard graph of 7me-GMP. For the ELISA based detection and quantification of 7me-GMP, competitive ELISA Kit (RayBiotech, EIA-m7G) was used and standard sigmoidal graph was prepared using 7me-GMP with a concentration range from 200 μg/mL to 2 pg/mL. **(B)** The interpolated values were converted to antilog for obtaining the exact concentration of 7me-GMP in each nucleotide sample of control and knockdown cell lines using GraphPad Prism software. B, absorbance values of antibody-binding response in the sample; B_0_ absorbance values of the blank (B); All data represent mean ± S.E. (error bars) from five biologically independent experiments (n = 5), each having 3 replicates.


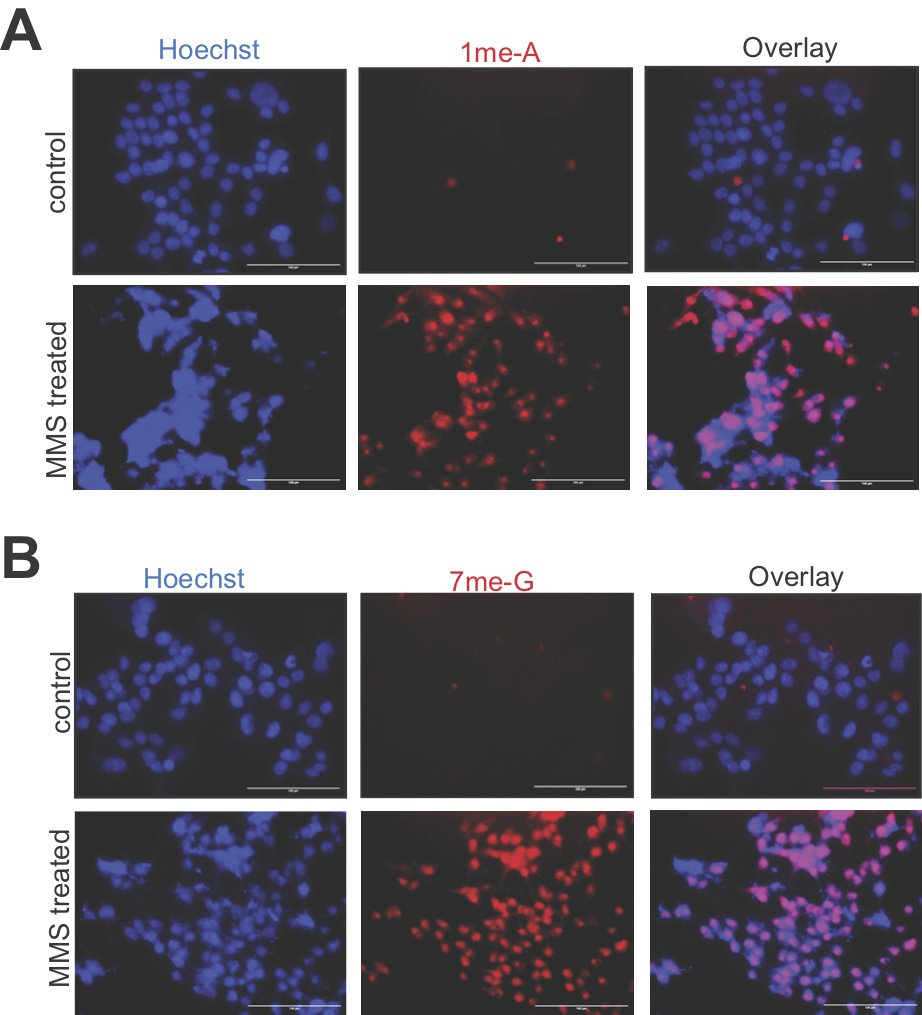


**Figure S14.** Evaluation of specificity of antibodies by immunofluorescence detection of 1me-AMP and 7me-GMP. 1me-AMP and 7me-GMP levels (red) were determined by immunofluorescence using the antibody against the lesions in untreated (control) or MMS treated (400 μM for 48 h) MCF7 cells. The permeabilized cells were stained using primary antibodies against 1me-A (rabbit) or 7meG (rabbit) (1: 250 dilution) and secondary antibody Alexa-Fluor 633 goat anti-rabbit (1:1000 dilution). Nuclei were stained using Hoechst 33258 is shown (blue). Presented results correspond to a representative experiment out of three independent ones. Scale bar: 100 μm.
